# Algebraic formulas for first-passage times of Markov processes in the linear framework

**DOI:** 10.1007/s11538-025-01524-z

**Published:** 2025-10-14

**Authors:** Kee-Myoung Nam, Jeremy Gunawardena

**Affiliations:** 1 Department of Systems Biology, Harvard Medical School, 200 Longwood Ave., Boston, MA 02115 USA; 2 Department of Molecular, Cellular and Developmental Biology, Yale University, 260 Whitney Ave., New Haven, CT 06511 USA; 3 Department of Medicine and Life Sciences, Pompeu Fabra University, Dr. Aiguader 80, Barcelona, 08003 Spain

**Keywords:** Labelled directed graphs, Laplacian matrix, Rational algebraic functions, First-passage times, Matrix-Tree theorems

## Abstract

The linear framework is an approach to analysing biochemical systems based on directed graphs with labelled edges. When applied to individual molecular systems, graph vertices correspond to system states, directed edges to transitions, and edge labels to transition rates. Such a graph specifies the infinitesimal generator of a continuous-time Markov process. The master equation of this Markov process, which describes the forward evolution of vertex probabilities, is a linear differential equation, after which the framework is named, whose operator is the Laplacian matrix of the graph. The Matrix-Tree theorem, when applied to this Laplacian matrix, allows the steady-state probabilities of the Markov process to be expressed as rational algebraic functions of the transition rates. This capability gives algebraic access to problems that have otherwise been treated by approximations or numerical simulations, and enables theorems to be proved about biochemical systems that rise above their underlying molecular complexity. Here, we extend this capability from the steady state to the transient regime. We use the All-Minors Matrix-Tree theorem to express the moments of the conditional first-passage time distribution, and the corresponding splitting probabilities, as rational algebraic functions of the transition rates. This extended capability brings many new biological problems within the scope of the linear framework.

## Introduction

In previous work, we introduced a graph-theoretic “linear framework” for analysing biochemical systems (Gunawardena 2012; Mirzaev and Gunawardena 2013); for a recent overview, see Nam et al. (2022). The framework is based on finite, simple, directed graphs with labelled edges and no self-loops. Given such a graph *G*, the vertices of *G*, $\mathcal {V}(G) = \left\{ 1, \dotsc , N \right\}$, typically represent biochemical components; the directed edges, $\mathcal {E}(G) \subseteq \mathcal {V}(G) \times \mathcal {V}(G)$, denoted $i \rightarrow j$, represent reactions between the corresponding components; and the edge labels, denoted $\ell (i \rightarrow j)$, represent positive reaction rates, with units of (time)$^{-1}$. The edge labels may include terms that describe interactions between the graph and its environment, such as the concentration of a binding ligand, and this allows typical biochemical nonlinearities to be accommodated.

A linear framework graph gives rise to a linear dynamics, from which the framework gets its name. The simplest way to describe this dynamics is to consider each edge as a chemical reaction obeying mass-action kinetics with the corresponding edge label as the rate constant. This yields a system of differential equations for the concentrations of the vertices, $\textbf{x}(t) = \left( x_1(t), \dotsc , x_N(t) \right) ^\textrm{T}$, where $^\textrm{T}$ denotes transpose. Since an edge has only one source vertex, these equations are linear and can be expressed in matrix form as

1 $$\begin{aligned} \frac{d\textbf{x}}{dt} = \mathcal {L}(G) \, \textbf{x}\,. \end{aligned}$$

Here, $\mathcal {L}(G)$ is the *Laplacian matrix* of the graph *G* (Chung 1997). Under appropriate scalings, Laplacian matrices correspond to discrete versions of the Laplacian differential operator (Chung 1997), so that Eqn. 1 may be thought of as a discretised diffusion equation. Since material is neither created nor destroyed by the dynamics, there is a conservation law,

$$\begin{aligned} x_1(t) + \cdots + x_N(t) = x_{\text {tot}} \,. \end{aligned}$$

This equation corresponds to the column sums of $\mathcal {L}(G)$ being zero, $\textbf{1}^\textrm{T}\, \mathcal {L}(G) = \textbf{0}^\textrm{T}$, where $\textbf{0}$ and $\textbf{1}$ denote the all-zero and all-one column vectors of the appropriate dimension, respectively. (Synthesis and degradation can be accommodated (Gunawardena 2012; Mirzaev and Bortz 2015) but are not considered here.) If we unwrap the definition of $\mathcal {L}(G)$, we see that the matrix entries are given by,

2 $$\begin{aligned} \begin{aligned} \mathcal {L}(G)_{i,j} = {\left\{ \begin{array}{ll} 0 & \text{ if } \,\,i \not = j \,\,\text{ and } \,\,j \not \rightarrow i \\ \ell (j \rightarrow i) & \text{ if } \,\, i \not = j\,\, \text{ and } \,\,j \rightarrow i \\ -\sum _{k:\, j \rightarrow k}{\ell (j \rightarrow k)} & \text{ if }\,\, i = j \,. \end{array}\right. } \end{aligned} \end{aligned}$$

**Fig. 1:**
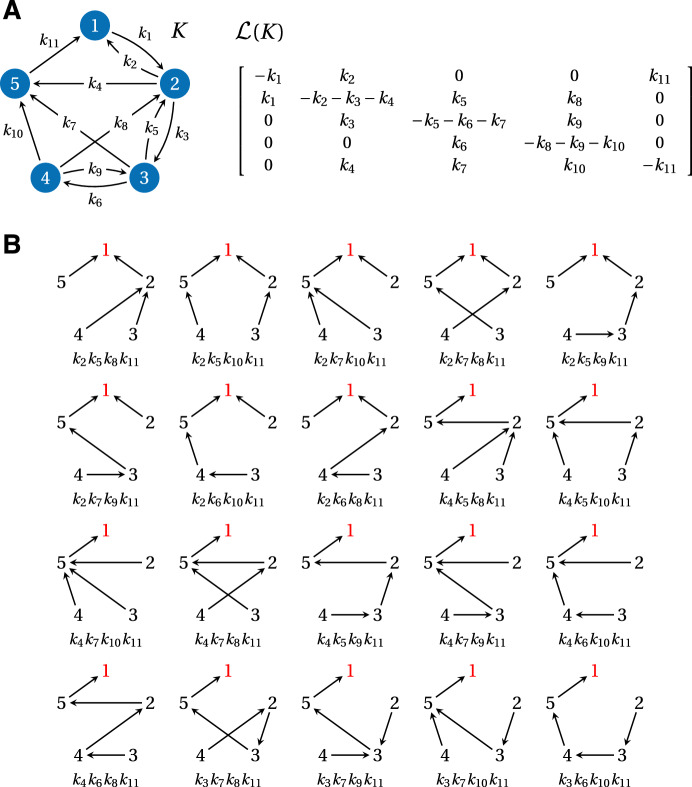
(**A**) An example linear framework graph, *K*, on five vertices and its Laplacian matrix ${{\mathcal {L}}}(K)$. This graph also appears in Nam and Gunawardena (2023, Fig. 2) but with a different labelling. (**B**) The 20 spanning trees of *K* that are rooted at vertex 1 (red), and their corresponding products of edge labels

An example linear framework graph and its associated Laplacian matrix are shown in Fig. 1A.

Two biochemical settings have been studied within the linear framework. In the first setting, the system is a macroscopic bulk mixture of biochemical components interacting through chemical reactions. Here, the variables correspond to the concentrations of the components, which evolve deterministically over time, as described by the Laplacian dynamics in Eqn. 1. A typical example is a network of enzymes and substrates; the linear framework was originally introduced to analyse such systems (Gunawardena 2012). For related applications in the macroscopic setting, see Dasgupta et al. (2014); Nam et al. (2020); Yordanov and Stelling (2018, 2020); and for a review, see Gunawardena (2014).

In the second setting, the system is a microscopic one, typically consisting of an individual macromolecule, such as a gene. The macromolecule evolves stochastically among a collection of states, partly through interactions with external components in the surrounding thermal bath. The vertices of the graph represent the states of the macromolecule, the edges represent transitions among these states, and the edge labels represent transition rates. The components in the environment are often assumed to be present in such abundance, in thermodynamic “reservoirs”, that their concentrations can be treated as constant during interactions with the graph. In this case, the edge labels are constants. This specification gives rise to a finite-state, continuous-time, time-homogeneous Markov process, *X*(*t*). Each variable, $x_i(t)$, in Eqn. 1 corresponds to the probability of the system occupying state *i* at time *t*; and there is an edge $i \rightarrow j$ if, and only if, the infinitesimal transition rate from *i* to *j* is positive,

3 $$\begin{aligned} \lim _{h \rightarrow 0}{\frac{\Pr {\left[ X(t + h) = j \mid X(t) = i \right] }}{h}} > 0 \,, \end{aligned}$$

and this positive rate is the corresponding edge label, $\ell (i \rightarrow j)$. Any finite-state, time-homogeneous Markov process for which the infinitesimal transition rates are well-defined can be described by a linear framework graph in this way (Mirzaev and Gunawardena 2013). (In some probability textbooks (Anderson 1991; Norris 1997), the infinitesimal generator of the Markov process is $\mathcal {L}(G)^\textrm{T}$ and is called the “*Q*-matrix.”) Gene regulation is a biological context in which the microscopic interpretation of the linear framework has been particularly useful (Ahsendorf et al. 2014; Estrada et al. 2016; Biddle et al. 2019; Park et al. 2019; Martinez-Corral et al. 2024; Nasser et al. 2024); for a review, see Wong and Gunawardena (2020).

In the microscopic interpretation, the Laplacian dynamics in Eqn. 1 is the *master equation*, or *Kolmogorov forward equation*, associated with *X*(*t*) (van Kampen 2007), which describes the deterministic time evolution of the probability distribution on the states in the process. It is an interesting fact that both the macroscopic and microscopic contexts can be described by the same Laplacian dynamics in Eqn. 1. In this paper, we will focus on the microscopic setting, so that $x_i(t)$ will denote probability rather than concentration, with $x_{\text {tot}} = 1$. We will accordingly be studying Markov processes from a graph-theoretic perspective.

Up to now, the focus of the linear framework has been on the steady state, which is appropriate for the timescale separations that are frequently used in analysing biochemical systems (Gunawardena 2014). It follows from Eqn. 1 that the steady state, $\textbf{x}^*$, lies in the kernel of $\mathcal {L}(G)$. A basis for $\ker \mathcal {L}(G)$ can be described in terms of the edge labels of *G* by the Matrix-Tree theorem (MTT) (Gunawardena 2012; Mirzaev and Gunawardena 2013); see Theorem 1. When *G* is strongly connected, meaning that there is a directed path of edges from each vertex to every other vertex in *G*, then (Gunawardena 2012),

$$\begin{aligned} \dim {\ker {\mathcal {L}(G)}} = 1 \,. \end{aligned}$$

The MTT shows that a canonical basis vector, $\varvec{\rho }(G) \in \ker {\mathcal {L}(G)}$, is given by

4 $$\begin{aligned} \rho _i(G) = \sum _{F \in \Phi _{\{i\}}(G)}{\left( \prod _{j \rightarrow k \in F}{\ell (j \rightarrow k)} \right) } \,, \end{aligned}$$

where $\Phi _{\{i\}}(G)$ is the set of *spanning trees* of *G* that are *rooted* at *i*. A spanning tree, *F*, of *G* is a subgraph of *G* which includes each vertex (“spanning”), has no cycles if edge directions are ignored (“tree”), and has exactly one outgoing edge from each vertex except one, which has no outgoing edges; this special vertex is the *root* of *F*. For instance, the graph in Fig. 1A, which is strongly connected, has 20 spanning trees that are rooted at the vertex 1, each of which contributes a product of four edge labels to the sum in Eqn. 4 (Fig. 1B). We can obtain the steady-state probability of vertex *i* from Eqn. 4 by normalising to the total,

5 $$\begin{aligned} x_i^* = \frac{\rho _i(G)}{\rho _1(G) + \cdots + \rho _N(G)} \,. \end{aligned}$$

The case of a general, non-strongly connected graph is discussed in Mirzaev and Gunawardena (2013). Importantly, Eqns. 4 and 5 are symbolic in the edge labels and do not require the numerical values of the transition rates to be known.

Since the Laplacian dynamics in Eqn. 1 is linear, the steady state is an eigenvector for the zero eigenvalue. This eigenvector can be readily obtained from ${{\mathcal {L}}}(G)$ but the resulting determinant formulas contain alternating signs. The MTT is important because it accounts for the remarkable cancellations that allow each $\rho _i(G)$ to be expressed as a *manifestly positive* polynomial in the edge labels (Eqn. 4). (The distinction here is that although the polynomial $a^2 - 2ab + b^2 = (a-b)^2$ is always positive when *a* and *b* are positive and unequal, it is not a sum of monomials with positive coefficients, which is “manifestly” positive.) Indeed, Eqn. 4 yields a sum of monomials whose coefficients are all positive integers, in this case the same integer, 1. Eqn. 5 then shows that steady-state probabilities are also rational algebraic functions of the edge labels with the same property. Positivity would be expected for the functional dependency of the probability on the edge labels but the manifest (integral) positivity is an additional mathematical feature. It arises because the steady-state probabilities of a Markov process are not merely probabilities. They are also governed by an underlying combinatorial structure, derived from the spanning trees of the corresponding linear framework graph, as expressed by the MTT (Eqn. 4). Manifest positivity also arises in other contexts; see Williams (2022) for more discussion. The existence within the steady state of an underlying combinatorial structure, in the form of spanning trees, from which the manifest positivity arises, is a mathematical feature that has been central to the linear framework.

Another significant consequence of the MTT that emerges from Eqn. 4 is that steady-state probabilities can be calculated irrespective of the thermodynamic context. If the graph can reach a steady state of thermodynamic equilibrium, so that *detailed balance*, or the *cycle condition*, is satisfied, then Eqn. 5 reduces to the standard prescription of equilibrium statistical mechanics, with the denominator corresponding to the partition function for the grand canonical ensemble (Gunawardena 2012; Ahsendorf et al. 2014; Estrada et al. 2016). However, Eqns. 4 and 5 hold equally well in a steady state away from thermodynamic equilibrium. The linear framework thereby provides a restricted context in which non-equilibrium statistical mechanics is exactly solvable. This capability has been exploited in several recent applications (Estrada et al. 2016; Biddle et al. 2019; Park et al. 2019; Martinez-Corral et al. 2024); for a review in the context of gene regulation, see Wong and Gunawardena (2020).

The use of graph-theoretic methods in stochastic thermodynamics goes back to the work of Hill (1966) and Schnakenberg (1976) and has been resurrected more recently; see, for example, Andrieux and Gaspard (2007); Murugan et al. (2014); Owen and Horowitz (2023); Dal Cengio et al. (2023); Qureshi et al. (2023). For biochemical systems, the graph theory approach of the linear framework allows appropriate molecular details to be left unspecified, by imposing purely topological requirements, while the rational algebraic access through Eqn. 5, which does not require parameter values to be known, has allowed rigorous theorems to be proved that rise above the underlying molecular complexity (Wong et al. 2018; Biddle et al. 2021; Martinez-Corral et al. 2024).

The linear framework has been successful in analysing systems at steady state but it had long been thought that it could not be extended to the transient regime, during which the system is relaxing to a steady state. It seemed that this regime could only be accessed through the eigenvalues of $\mathcal {L}(G)$. The main contribution of the present paper is to show that this is not the case. It is possible to express certain transient quantities, such as the moments of the *first-passage time* (FPT) distribution, as manifestly positive rational algebraic functions of the edge labels. This capability opens up a large class of new problems that can potentially be addressed in a similar way to the applications described above.

The FPT from one vertex, *i*, to another, *j*, is the random variable measuring the time taken by a trajectory of the associated Markov process to reach *j* for the first time, when started from *i*. FPTs have been used to quantify a variety of observables in molecular biophysics: the completion time of an enzymatic turnover (Kou et al. 2005; Shaevitz et al. 2005; Kolomeisky and Fisher 2007; Chemla et al. 2008; Garai et al. 2009; Bel et al. 2010; Moffitt et al. 2010; Moffitt and Bustamante 2014), the probability and speed with which an enzyme can choose between correct and incorrect reaction pathways (Banerjee et al. 2017; Cui and Mehta 2018; Mallory et al. 2019; Wang et al. 2021), the speed with which a gene alternates between different regulatory states (Lammers et al. 2020; Alamos et al. 2023; Lammers et al. 2023), the time taken by a regulated molecule to cross an abundance threshold (Dal Co et al. 2017; Ghusinga et al. 2017; Gupta et al. 2018; Ham et al. 2024), and the rate of templated copolymer growth (Qureshi et al. 2023; Guntoro et al. 2025). However, despite their broad usefulness in biology, FPTs have usually been calculated numerically or by *ad hoc* methods for special cases (Bel et al. 2010), or by more general methods for which closed-form solutions are not readily found (Shaevitz et al. 2005; Chemla et al. 2008). Even when rational functions emerge in such treatments, their manifest positivity may not always be visible (Bel et al. 2010). The use of Terrell Hill’s method for calculating mean FPTs (Hill 1988), as in Qureshi et al. (2023); Guntoro et al. (2025), is an exception to which we will return below.

In this paper, we introduce a systematic graph-theoretic treatment of FPTs for Markov processes, and thereby extend the scope of the linear framework from the steady state to the transient regime. To do this, we exploit a generalisation of the MTT, the *All-Minors Matrix-Tree theorem* (AMMTT), stated here as Theorem 1. The AMMTT uses *spanning forests*, which generalise the spanning trees of the classical MTT described above. The AMMTT enables us to provide closed-form solutions for the moments of any FPT distribution, either unconditional or conditional, along with related quantities such as splitting probabilities, as manifestly positive rational algebraic functions of the edge labels.

The AMMTT has been used to derive formulas for mean FPTs (Chebotarev 2007; Pitman and Tang 2018) and for splitting probabilities (Pitman and Tang 2018) in discrete-time Markov chains, but not, to our knowledge, for higher moments and conditional FPTs of the continuous-time Markov processes studied here.

Generalisations of the classical work on FPTs of Hill (1988) and of Kac (1947) can be shown to follow from the results presented here. For reasons of space, we discuss these connections in a sequel to the present paper (Nam and Gunawardena 2025). The main application of our results so far has been to the analysis of a tradeoff between accuracy and speed in the CRISPR–Cas9 system, which will also be presented elsewhere (Nam et al. 2025).

We begin with some preliminary definitions and results; a summary of the various symbols and notation that we use is also provided in Table 1. The AMMTT is introduced in Theorem 1. We deduce an important consequence of this in Proposition 2, which shows how the inverse of a principal submatrix of the Laplacian matrix may be calculated in terms of spanning forests. This technical result is a key ingredient in what follows. We then focus on the special case of graphs in which the target vertices for FPTs are the “terminal” vertices in the graph, in a sense explained below. This special case allows a differential equation to be formulated for the time evolution of the probabilities of reaching the target vertices (Eqn. 34). The Laplace transform of this *adjoint master equation* allows the moments of the appropriate FPT distribution to be calculated in a standard way (Eqn. 42). This moment calculation requires the inverse of a principal submatrix of the Laplacian matrix (Eqn. 44), which Proposition 2 describes in terms of spanning forests (Eqn. 47). It is then straightforward to deduce our main results: Theorem 4 for the splitting probabilities, Theorem 5 for the moments of the conditional FPT to a specific target vertex, and Theorem 6 for the moments of the unconditional FPT to any target vertex. We then explain how FPTs may be calculated in a general graph by modifying the graph so that it falls into the special case on which the previous analysis was based. Finally, we work through some examples, of pipeline graphs (Fig. 4) and of a butterfly graph (Fig. 5), both taken from the literature, to illustrate how the methods introduced here can be used in practice.

The work described here was begun in K-MN’s Harvard PhD thesis (Nam 2021), under the supervision of JG. The present paper uses a more direct proof strategy and fills in some details that were omitted in Nam (2021). An overview of this material, without proofs but with examples, appeared in Nam and Gunawardena (2023), which should be consulted for further background.

## Results

### Background and preliminaries

#### Graphs and spanning forests

We begin by establishing some notation and terminology. See the Table in the Appendix for a summary. We will continue to use the notation in the Introduction, with decorations where necessary, as in $i \rightarrow _G j$, to specify which graph, *G*, is intended. We assume that all graphs are connected when edge directions are ignored, so that they do not break up into separated pieces.

Given $i \in \mathcal {V}(G)$, let $\textrm{V}_{\text {out}}(i) \subseteq \mathcal {V}(G)$ denote the set of vertices to which *i* has an edge, $\textrm{V}_{\text {out}}(i) = \left\{ j \in \mathcal {V}(G) : i \rightarrow j \right\}$, and let $\textrm{V}_{\text {in}}(i) \subseteq \mathcal {V}(G)$ denote the set of vertices which have an edge to *i*, $\textrm{V}_{\text {in}}(i) = \left\{ j \in \mathcal {V}(G) : j \rightarrow i \right\}$.

Let $A, B \subseteq \mathcal {V}(G) = \left\{ 1, \dotsc , N \right\}$ be vertex subsets. We denote the size of *A* by $\# A$ and the complement of *A* by ${\overline{A}}= \mathcal {V}(G) \setminus A$. Given a matrix $\textbf{M}\in \mathbb {R}^{N \times N}$, we denote by $\textbf{M}_{[A,B]} \in \mathbb {R}^{\# A \times \# B}$ the submatrix consisting of the rows indexed by *A* and the columns indexed by *B*. We use $\textbf{I}$ to denote the identity matrix, leaving its dimensions to be inferred from context.

Let *H* be a subgraph of *G*. The *weight* of *H*, denoted *w*(*H*), is the product of the edge labels in *H*,

$$\begin{aligned} w(H) = \prod _{i \rightarrow j \in H}{\ell (i \rightarrow j)} \,. \end{aligned}$$

We follow the standard convention that empty sums evaluate to zero and empty products evaluate to one. It follows that the weight of an edgeless graph is one. If $\mathcal {H}$ is a set of subgraphs of *G*, then $w(\mathcal {H})$ is the sum of the weights of the subgraphs in $\mathcal {H}$,

$$\begin{aligned} w(\mathcal {H}) = \sum _{H \in \mathcal {H}}{w(H)} \,. \end{aligned}$$

A subgraph *F* of *G* is a *spanning forest* of *G* if it contains every vertex in *G* (“spanning”), it contains no cycles of edges when edge directions are ignored (“forest”), and each vertex has at most one outgoing edge (which orients the forest). Since *F* has no cycles, it must have at least one vertex with no outgoing edges. Such vertices are the *roots* of *F*; let $\varnothing \not = \mathcal {R}(F) \subseteq \mathcal {V}(G)$ denote the set of all roots of *F*. If $\mathcal {R}(F) = \{i\}$, then *F* is a *spanning tree rooted at i*, as defined in the Introduction. Note that a spanning forest *F* is a disjoint union of $\#\mathcal {R}(F)$ trees, and must have exactly $N - \#\mathcal {R}(F)$ edges. We denote the set of all spanning forests of *G* rooted at *A* by $\Phi _A(G)$.

Given two vertices, $i, j \in \mathcal {V}(G)$, we say that *i* *leads to* *j*, denoted $i \leadsto j$, if there is a directed path of edges from *i* to *j*: $i = i_1 \rightarrow \cdots \rightarrow i_k = j$. There is always a trivial path from any vertex to itself, so that $i \leadsto i$. Furthermore, if *F* is a spanning forest of *G*, then any $i \in \mathcal {V}(G)$ has a unique directed path in *F*, with no repeated vertices, to exactly one root, namely the root of the tree containing *i*.

To accompany the vertex subsets $\textrm{V}_{\text {out}}(i)$ and $\textrm{V}_{\text {in}}(i)$ coming from edges adjoining $i \in \mathcal {V}(G)$, as defined above, we denote by $\mathcal {V}_{\text {out}}(i)$ and $\mathcal {V}_{\text {in}} (i)$ the corresponding vertex subsets for directed paths that lead from *i*, $\mathcal {V}_{\text {out}}(i) = \{ j \in \mathcal {V}(G) : i \leadsto j \}$, and lead to *i*, $\mathcal {V}_{\text {in}} (i) = \{ j \in \mathcal {V}(G) : j \leadsto i \}$, respectively. Note that $i \not \in \textrm{V}_{\text {out}}(i)$ but $i \in \mathcal {V}_{\text {out}}(i) \not = \varnothing$; similarly, $i \not \in \textrm{V}_{\text {in}}(i)$ but $i \in \mathcal {V}_{\text {in}} (i) \not = \varnothing$. It is possible that one of $\textrm{V}_{\text {out}}(i)$ or $\textrm{V}_{\text {in}}(i)$ is empty but not both, unless *G* consists solely of the vertex *i*.

Given $A, B \subseteq \mathcal {V}(G)$ with $\# A = \# B$, define $\Phi _{B \rightarrow A}(G) \subseteq \Phi _A(G)$ as the set of spanning forests of *G* rooted at *A* in which each vertex in *B* has a path to a *distinct* root in *A*. So, $\Phi _{\{1,2\} \rightarrow \{3,4\}}(G)$ is the set of all spanning forests of *G* rooted at $\left\{ 3,4 \right\}$ in which either $1 \leadsto 3$, $2 \leadsto 4$ or $1 \leadsto 4$, $2 \leadsto 3$. Since every root has a trivial path to itself, it follows that,

6 $$\begin{aligned} \Phi _{A \rightarrow A}(G) = \Phi _A(G) \qquad \text {for all }\, A \subseteq \mathcal {V}(G) \,. \end{aligned}$$

If $A = \{j\}$, then each $F \in \Phi _A(G)$ is a spanning tree and every vertex has a path to *j*. Hence, $\Phi _{\{i\} \rightarrow \{j\}}(G) = \Phi _{\{j\}}(G)$ for all $i, j \in \mathcal {V}(G)$. A spanning forest $F \in \Phi _{B \rightarrow A}(G)$ induces a bijection, $\chi _F : B \rightarrow A$, where $\chi _F(i) \in A$ is the unique root to which there is a path from *i* in *F*. Since a root always has a path to itself, $\chi _F(i) = i$ for all $i \in A \cap B$. In particular, if $A = B$, then $\chi _F : A \rightarrow A$ is the identity for all $F \in \Phi _{A \rightarrow A}(G) = \Phi _A(G)$ (Eqn. 6).

We say that *i* is *strongly connected to j* if $i \leadsto j$ and $j \leadsto i$. Strong connectedness is an equivalence relation on $\mathcal {V}(G)$, and the corresponding equivalence classes are the *strongly connected components* (SCCs) of *G*. We denote the set of SCCs of *G* by $\mathcal {C}(G)$. Given $C, D \in \mathcal {C}(G)$, we say that *C* *precedes* *D*, denoted $C \prec D$, if $i \leadsto j$ for some $i \in C$ and some $j \in D$. Since *C* and *D* are each strongly connected, this implies that $i \leadsto j$ for any $i \in C$ and any $j \in D$. In particular, if $C \prec D$ and $D \prec C$, then $C = D$. Hence, the relation $C \preceq D$, defined as $C = D$ or $C \prec D$, defines a partial order on $\mathcal {C}(G)$. The maximal elements under this partial order are the *terminal SCCs* of *G*, collectively denoted by $\mathcal {T}(G) \subseteq \mathcal {C}(G)$. Note that $\textrm{V}_{\text {out}}(i) = \varnothing$ if, and only if, $\{i\}$ is a singleton terminal SCC.

The terminal SCCs determine the minimal number of roots in a spanning forest. Suppose that *G* has *T* terminal SCCs, $C_1, \dotsc , C_T$. Let $\mathcal {Q}(G) = C_1 \cup \cdots \cup C_T$ denote all the vertices within the terminal SCCs, and let $\mathcal {N}(G) = \mathcal {V}(G) \setminus \mathcal {Q}(G)$ be all the vertices within the non-terminal SCCs. Since there can be no directed paths between terminal SCCs, any spanning forest *F* of *G* must have at least one root in each terminal SCC. Hence, $\#\mathcal {R}(F) \ge T$. If *G* is strongly connected, so that $T = 1$, then it is clear that the preceding inequality is sharp. If *G* is not strongly connected, then, because *G* is connected, it must be that $\mathcal{N}(G) \not = \varnothing$. Each $i \in \mathcal {N}(G)$ must lead to some $j \in \mathcal {Q}(G)$ and, because *G* is connected, every $j \in \mathcal {Q}(G)$ must have some vertex in $\mathcal {N}(G)$ that leads to it. It follows that, provided $A \subseteq \mathcal {V}(G)$ contains at least one vertex from each terminal SCC, so that $A \cap C_k \not = \varnothing$ for $1 \le k \le T$, then there is a spanning forest *F* with $\mathcal {R}(F) = A$. In particular, there is always a spanning forest with exactly *T* roots, so that the inequality $\#\mathcal {R}(F) \ge T$ is sharp.

#### The All-Minors Matrix-Tree theorem

The Matrix-Tree theorem (MTT) allows the steady-state probabilities of a graph to be expressed in terms of the edge labels, as shown by Eqns. 4 and 5. Here, we introduce the more general All-Minors Matrix-Tree theorem (AMMTT), which we use to express FPTs in the same way. This class of theorems reveals that Laplacian matrices, $\mathcal {L}(G)$, have remarkable properties: the determinants of submatrices, or the minors, of $\mathcal {L}(G)$ exhibit extensive sign cancellations and can be expressed in terms of the weights of spanning forests of *G*. We saw this for steady-state probabilities and spanning trees in Eqn. 4.

A bijection, $\sigma : A \rightarrow A$, is a *permutation* on *A*, whose sign, ${{\,\textrm{sgn}\,}}(\sigma )$, is given by $(-1)^p$, where *p* is the number of transpositions in any decomposition of $\sigma$ into a product of transpositions (Herstein 1975). Recall that $\mathcal {L}(G)_{[{\overline{B}},{\overline{A}}]}$ is the submatrix of $\mathcal {L}(G)$ in which the rows indexed by *B* and the columns indexed by *A* have been removed. If $\#A = \#B$, then the determinant of such a submatrix is a *minor* of $\mathcal {L}(G)$.

##### Theorem 1

(All-Minors Matrix-Tree theorem) Let *G* be a graph, and let $\varnothing \not = A, B \subsetneq \mathcal {V}(G)$ be proper, non-empty vertex subsets of the same size, $\# A = \# B = k$. Write *A* and *B* in ascending order as $A = \left\{ a_1< \cdots < a_k \right\}$ and $B = \left\{ b_1< \cdots < b_k \right\}$ and let $\eta : A \rightarrow B$ be the bijection $\eta (a_i) = b_i$ for $i = 1, \dotsc , k$. Then,

7 $$\begin{aligned} \det {\mathcal {L}(G)_{[{\overline{B}},{\overline{A}}]}} = (-1)^{N - k + \sum _{a \in A}{a} + \sum _{b \in B}{b}} \sum _{F \in \Phi _{B \rightarrow A}(G)}{{{\,\textrm{sgn}\,}}(\chi _F \circ \eta ) \, w(F)} \,, \end{aligned}$$

where $\chi _F : B \rightarrow A$ is the bijection defined after Eqn. 6.

If we take $A = \left\{ i \right\}$ and $B = \left\{ j \right\}$, the AMMTT reduces to the MTT (Mirzaev and Gunawardena 2013), from which Eqn. 4 is derived. The MTT has a long history that goes back to the 19th century work of Kirchhoff—see Mirzaev and Gunawardena (2013) for details—but the first report of the AMMTT is due to Fiedler and Sedláček (1958) in the Czech mathematical literature; subsequent proofs were given by Chaiken (1982) and Moon (1994).

**Fig. 2:**
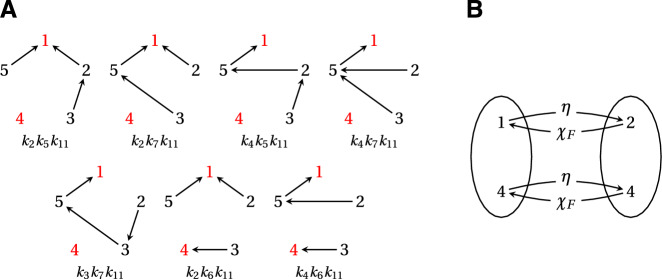
(**A**) The seven spanning forests in $\Phi _{\{2,4\} \rightarrow \{1,4\}}(K)$, where *K* is the example graph in Fig. 1A, with their corresponding weights. Roots are shown in red. (**B**) Schematic of the maps between $\{1,4\}$ and $\{2,4\}$ for each forest *F* in panel **A**. In this case, $\chi _F$ is the same for all *F* and is the inverse of $\eta$

As an example of Theorem 1 in action, consider the graph, *K*, in Fig. 1A, whose Laplacian matrix, $\mathcal {L}(K)$, is also shown there. The submatrix $\mathcal {L}(K)_{[\overline{\{2,4\}},\overline{\{1,4\}}]}$ is given by

8 $$\begin{aligned} \left[ \begin{array}{ccc} k_2 & 0 & k_{11} \\ k_3 & -k_5 - k_6 - k_7 & 0 \\ k_4 & k_7 & -k_{11} \\ \end{array} \right] \,. \end{aligned}$$

According to Theorem 1, the minor, $\det {\mathcal {L}(K)_{[\overline{\{2,4\}}, \overline{\{1,4\}}]}}$, may be calculated in terms of the spanning forests in $\Phi _{\{2,4\} \rightarrow \{1,4\}}(K)$, which are enumerated in Fig. 2A. Since vertex 4 is common to both $\{1,4\}$ and $\{2,4\}$, the map $\chi _F: \{2,4\} \rightarrow \{1,4\}$ is the same for all $F \in \Phi _{\{2,4\} \rightarrow \{1,4\}}(K)$, and this map is also the inverse of the map $\eta : \{1,4\} \rightarrow \{2,4\}$ defined in Theorem 1 (Fig. 2B). It follows that ${{\,\textrm{sgn}\,}}{(\chi _F \circ \eta )} = 1$ for all $F \in \Phi _{\{2,4\} \rightarrow \{1,4\}}(K)$. Furthermore, $N - k = 5 - 2 = 3$ and $(1 + 4) + (2 + 4) = 11$ combine to yield a sign of $(-1)^{14} = 1$. It follows from Theorem 1 that,

$$\begin{aligned} \det {\mathcal {L}(K)_{[\overline{\{2,4\}}, \overline{\{1,4\}}]}} = k_{11} \left( k_2 k_5 + k_2 k_7 + k_4 k_5 + k_4 k_7 + k_3 k_7 + k_2 k_6 + k_4 k_6 \right) , \end{aligned}$$

which may easily be confirmed from Eqn. 8.

As a further example of how Theorem 1 works, consider the extreme case in which $A = \overline{\{i\}}$ and $B = \overline{\{j\}}$ with $i \not = j$, so that $k = N-1$. Then,

$$\begin{aligned} \mathcal {L}(G)_{[{\overline{B}},{\overline{A}}]} = \mathcal {L}(G)_{j,i}, \end{aligned}$$

so that (Eqn. 2),

9 $$\begin{aligned} \begin{aligned} \det {\mathcal {L}(G)_{[{\overline{B}},{\overline{A}}]}} = {\left\{ \begin{array}{ll} \ell (i \rightarrow j) & \text{ if }\,\, i \rightarrow j \\ 0 & \text{ if }\,\, i \not \rightarrow j. \end{array}\right. } \end{aligned} \end{aligned}$$

To see what Theorem 1 says in this context, note that a spanning forest in $\Phi _{B \rightarrow A}(G)$ must contain only a single edge, which must evidently be $i \rightarrow j$, should the edge exist. Hence, if $i \not \rightarrow j$, then $\Phi _{B \rightarrow A}(G) = \varnothing$ and Theorem 1 correctly gives the second case in Eqn. 9. If, however, $i \rightarrow j$ does exist in *G*, then Theorem 1 says that $\det {\mathcal {L}(G)_{[{\overline{B}},{\overline{A}}]}}$ is given by

$$\begin{aligned} \left( -1 \right) ^{N - (N-1) + \sum _{a \in A}{a} + \sum _{b \in B}{b}} {{\,\textrm{sgn}\,}}{\left( \chi _F \circ \eta \right) } \, \ell (i \rightarrow j). \end{aligned}$$

We now examine how the two signs work out. First, note that the parity of $1 + \sum _{a \in A} a + \sum _{b \in B} b = 1 + 2 \left( 1 + \cdots + N \right) - i - j$ is the same as that of $1 - i - j$. As for the sign of $\chi _F \circ \eta$, assume that $i < j$ and let us write *A* and *B* out as a $2 \times (N-1)$ array of entries,

$$\begin{aligned} \begin{array}{cccccccccccl} A : & 1, & \dotsc , & i-1, & \underline{i}, & i+1, & \dotsc , & j-1, & j, & j+1, & \dotsc , & N \\ B : & 1, & \dotsc , & i-1, & i, & i+1, & \dotsc , & j-1, & \underline{j}, & j+1, & \dotsc , & N, \end{array} \end{aligned}$$

where $\underline{i}$ and $\underline{j}$ signify their absence from the array. It is clear that $\chi _F$ takes *i* to *j* and is the identity elsewhere, while from the array above, we see that $\eta$ takes *m* to $m-1$ if $i < m \le j$ and is the identity elsewhere. It follows that $\chi _F \circ \eta$ is the permutation,

$$\begin{aligned} \left( \begin{array}{ccccccccccc} 1 & \cdots & i-1 & i+1 & i+2 & \cdots & j-1 & j & j+1 & \cdots & N \\ 1 & \cdots & i-1 & j & i+1 & \cdots & j-2 & j-1 & j+1 & \cdots & N \end{array} \right) , \end{aligned}$$

which can be written in cycle notation as $(j, j-1, j-2, \dotsc , i+1)$. This cycle has length $j - i$, and therefore can be written as a product of $j - i - 1$ transpositions, so that

$$\begin{aligned} {{\,\textrm{sgn}\,}}{(\chi _F \circ \eta )} = \left( -1 \right) ^{j-i-1}. \end{aligned}$$

Accordingly, the total sign is

$$\begin{aligned} \left( -1 \right) ^{1-i-j+j-i-1} = \left( -1 \right) ^{-2i} = 1. \end{aligned}$$

Theorem 1 therefore tells us that, if $i \rightarrow j$ does exist in *G*, then $\det {\mathcal {L}(G)_{[{\overline{B}},{\overline{A}}]}} = \ell (i \rightarrow j)$, which agrees with the first case in Eqn. 9. We hope these examples illustrate the workings of Theorem 1.

#### Preliminary results

In this section, we state and prove some basic results that follow from Theorem 1, upon which we will rely throughout the paper.

We need some machinery to accomplish this. It will be convenient to define, for any finite, ordered set, $S = \left\{ s_1< \cdots < s_k \right\}$, the ordering bijection,

$$\begin{aligned} \theta _S : \left\{ 1, \dotsc , k \right\} \rightarrow S, \qquad \theta _S(i) = s_i \,. \end{aligned}$$

This bijection is helpful to keep track of the rows and columns of submatrices, which acquire a different indexing to those of the original matrix. Let $A \subseteq \mathcal {V}(G)$ be a subset with $\# A = k$, and let $i, j \in \left\{ 1, \dotsc , k \right\}$. Then the (*i*, *j*)-th entry of the $k \times k$ submatrix, $\mathcal {L}(G)_{[A, A]}$, corresponds to the $(\theta _A(i), \theta _A(j))$-th entry of $\mathcal {L}(G)$,

10 $$\begin{aligned} \left( \mathcal {L}(G)_{[A, A]} \right) _{i,j} = \mathcal {L}(G)_{\theta _A(i), \theta _A(j)} \,. \end{aligned}$$

Alternatively, if $i, j \in A$, then we can invert the correspondence in Eqn. 10 to see that,

11 $$\begin{aligned} \mathcal {L}(G)_{i,j} = \left( \mathcal {L}(G)_{[A, A]} \right) _{\theta _A^{-1}(i), \theta _A^{-1}(j)} \,. \end{aligned}$$

We now consider what happens when we re-index the vertices of *G* by a permutation, $\sigma : \mathcal {V}(G) \rightarrow \mathcal {V}(G)$. What this means is that the edge, $j \rightarrow _G i$, becomes the edge $\sigma (j) \rightarrow _{G^{\sigma }} \sigma (i)$ and retains the same label, $\ell (j \rightarrow _G i) = \ell (\sigma (j) \rightarrow _{G^{\sigma }} \sigma (i))$. It then follows from Eqn. 2 that,

12 $$\begin{aligned} \mathcal {L}(G)_{i,j} = \mathcal {L}(G^\sigma )_{\sigma (i), \sigma (j)} \,, \end{aligned}$$

so that the rows and columns of $\mathcal {L}(G)$ are permuted by $\sigma$ to form $\mathcal {L}(G^{\sigma })$. If we have the same subset $A \subseteq \mathcal {V}(G)$ as above, it will give rise under the permutation $\sigma$ to the subset $\sigma (A) \subseteq \mathcal {V}(G^{\sigma })$. If $i,j \in \sigma (A)$, then applying Eqn. 11 to $G^{\sigma }$, we see that,

13 $$\begin{aligned} \mathcal {L}(G^\sigma )_{i,j} = \left( \mathcal {L}(G^\sigma )_{[\sigma (A), \sigma (A)]} \right) _{\theta _{\sigma (A)}^{-1}(i), \theta _{\sigma (A)}^{-1}(j)} \,. \end{aligned}$$

Now let us return to choosing $i,j \in \{1, \dotsc , k\}$ and consider the sequence of bijections,which amount to a form of conjugation of $\sigma$ by $\theta _A$. Let us call the composition of these bijections $\tau _{\sigma , A}: \{1, \dotsc , k\} \rightarrow \{1, \dotsc , k\}$,

14 $$\begin{aligned} \tau _{\sigma ,A} = \theta _{\sigma (A)}^{-1}\left( \sigma \left( \theta _A \right) \right) \,. \end{aligned}$$

Applying Eqns. 10, 12 and 13 in sequence, we then see that,

15 $$\begin{aligned} \begin{aligned} \left( \mathcal {L}(G)_{[A, A]} \right) _{i,j}&= \mathcal {L}(G)_{\theta _A(i), \theta _A(j)} \\&= \mathcal {L}(G^{\sigma })_{\sigma (\theta _A(i)), \sigma (\theta _A(j))} \\&= \left( \mathcal {L}(G^\sigma )_{[\sigma (A), \sigma (A)]} \right) _{\tau _{\sigma ,A}(i), \tau _{\sigma ,A}(j)} \,. \end{aligned} \end{aligned}$$

The impact of the permutation $\sigma$ on spanning forests is less intricate. If $A \subseteq \mathcal {V}(G)$ and $F \in \Phi _A(G)$, then applying $\sigma$ to the vertices of *F* yields a re-indexed forest, $\sigma ^*(F) \in \Phi _{\sigma (A)}(G^\sigma )$, with the same weight, $w(\sigma ^*(F)) = w(F)$. If we have a second subset, $B \subseteq \mathcal {V}(G)$, with $\# B = \# A$, then the map, $\sigma ^* : \Phi _A(G) \rightarrow \Phi _{\sigma (A)}(G^\sigma )$, which is evidently a bijection, restricts to a bijection $\sigma ^* : \Phi _{B \rightarrow A}(G) \rightarrow \Phi _{\sigma (B) \rightarrow \sigma (A)}(G^\sigma )$. It follows that,

16 $$\begin{aligned} w(\Phi _{B \rightarrow A}(G)) = w(\Phi _{\sigma (B) \rightarrow \sigma (A)}(G^\sigma )) \,. \end{aligned}$$

Finally, we review some linear algebra. To derive the main results in this paper, we shall primarily invoke Theorem 1 in combination with Cramer’s rule. Recall that, if $\textbf{M}$ is an $N \times N$ matrix, then the *adjugate* of $\textbf{M}$, which we denote by ${{\,\textrm{adj}\,}}\textbf{M}$, is the $N \times N$ matrix with entries,

17 $$\begin{aligned} \begin{aligned} ({{\text {adj}\,}} {\textbf {M}})_{i,j} = (-1)^{i+j} \det {{\textbf {M}}_{[\overline{\{j\}}, \overline{\{i\}}]}} \,. \end{aligned} \end{aligned}$$

Cramer’s rule then states that,

18 $$\begin{aligned} \begin{aligned} {\textbf {M}}\, ({{\text {adj}\,}} {\textbf {M}}) = ({{\text {adj}\,}} {\textbf {M}}) \, {\textbf {M}}= (\det {\textbf {M}}) \, {\textbf {I}}\,. \end{aligned} \end{aligned}$$

It follows that, when $\det \textbf{M}\ne 0$, ${\textbf {M}}^{-1} = ({{\text {adj}\,}} {\textbf {M}}) / (\det {\textbf {M}})$.

This preparation leads us to one of the central technical results of the paper.

##### Proposition 2

Let *G* be a graph and let $\varnothing \ne A \subsetneq \mathcal {V}(G)$ be a subset of *k* vertices, where $0< k < N$. Then $\mathcal {L}(G)_{[{\overline{A}},{\overline{A}}]}$ is invertible if, and only if, $\Phi _A(G)$ is non-empty. Furthermore, if $\mathcal {L}(G)_{[{\overline{A}},{\overline{A}}]}$ is invertible, the inverse is given by,

$$\begin{aligned} \left( \mathcal {L}(G)_{[{\overline{A}},{\overline{A}}]} \right) ^{-1}_{i,j} = -\frac{w(\Phi _{A \cup \{\theta _{{\overline{A}}}(j)\} \rightarrow A \cup \{\theta _{{\overline{A}}}(i)\}}(G))}{w(\Phi _A(G))} \,, \end{aligned}$$

for $$i, j \in \left\{ 1, \dotsc , N-k \right\} $$.

##### Proof

The first statement follows by setting $A = B$ in Eqn. 7 and recalling Eqn. 6. To prove the second statement, we first show that it holds when $A = \left\{ N-k+1, \dotsc , N \right\}$, in which case $\theta _{{\overline{A}}}(i) = i$ for all $i = 1, \dotsc , N-k$. Then Eqn. 18 says that,

19 $$\begin{aligned} \left( \mathcal {L}(G)_{[{\overline{A}},{\overline{A}}]} \right) ^{-1} = \left( \frac{1}{\det \mathcal {L}(G)_{[{\overline{A}},{\overline{A}}]}}\right) {{\,\textrm{adj}\,}}\left( \mathcal {L}(G)_{[{\overline{A}},{\overline{A}}]}\right) \,, \end{aligned}$$

and Eqn. 17 tells us that,

20 $$\begin{aligned} {{\,\textrm{adj}\,}}\left( \mathcal {L}(G)_{[{\overline{A}},{\overline{A}}]}\right) _{i,j} = \left( -1 \right) ^{i+j} \det \mathcal {L}(G)_{[\overline{A \cup \{j\}}, \overline{A \cup \{i\}}]} \,. \end{aligned}$$

At this point, we can appeal to Theorem 1 to get the minor on the right as,

21 $$\begin{aligned} \left( -1 \right) ^{N-k-1+i+j+2\sum _{a \in A}{a}} \sum _{F \in \Phi _{A \cup \{j\} \rightarrow A \cup \{i\}}(G)}{{{\,\textrm{sgn}\,}}{(\chi _F \circ \eta )} \, w(F)} \,. \end{aligned}$$

Since $A = \left\{ N - k + 1, \dotsc , N \right\}$ and $i, j < N - k + 1$, the bijection $\eta : A \cup \{i\} \rightarrow A \cup \{j\}$ takes *i* to *j* and is the identity elsewhere,

$$\begin{aligned} \begin{aligned} \eta (q) = {\left\{ \begin{array}{ll} j & \text{ if }\,\, q = i \\ q & \text{ if }\,\, q \ne i\,. \end{array}\right. } \end{aligned} \end{aligned}$$

To determine the signs in Eqn. 21, the parity term contributes $(-1)^{N-k-1+i+j}$. As for the permutation term, for any forest $F \in \Phi _{A \cup \{j\} \rightarrow A \cup \{i\}}(G)$, $\chi _F$ must take *j* to *i* and be the identity elsewhere. Hence, $\chi _F \circ \eta$ is the identity on $A \cup \{i\}$ for each *F*. Therefore, the minor given by Eqn. 21 simplifies to,

$$\begin{aligned} \det {\mathcal {L}(G)_{[\overline{A \cup \{j\}}, \overline{A \cup \{i\}}]}} = \left( -1 \right) ^{N-k-1+i+j} w(\Phi _{A \cup \{j\} \rightarrow A \cup \{i\}}(G)) \,. \end{aligned}$$

We can combine this with Eqn. 20 to get,

$$\begin{aligned} {{\,\textrm{adj}\,}}\left( \mathcal {L}(G)_{[{\overline{A}},{\overline{A}}]}\right) _{i,j} = \left( -1 \right) ^{N-k-1} w(\Phi _{A \cup \{j\} \rightarrow A \cup \{i\}}(G)) \,. \end{aligned}$$

As for $\det {\mathcal {L}(G)_{[{\overline{A}},{\overline{A}}]}}$, Theorem 1 tells us that,

$$\begin{aligned} \det {\mathcal {L}(G)_{[{\overline{A}},{\overline{A}}]}} = \left( -1 \right) ^{N-k} w(\Phi _A(G)) \,. \end{aligned}$$

Incorporating these calculations into Eqn. 19, we obtain the required conclusion for the case where $A = \left\{ N-k+1, \dotsc , N \right\}$.

We now consider the case of a general subset, *A*, which may not be indexed as previously. Write *A* and ${\overline{A}}$ in ascending order, as $A = \left\{ a_1< \cdots < a_k \right\}$ and ${\overline{A}}= \left\{ {\overline{a}}_1< \cdots < {\overline{a}}_{N-k} \right\}$, and let $B = \left\{ N-k+1, \dotsc , N \right\}$. Let $G^\sigma$ be the graph obtained by re-indexing the vertices according to the permutation that maps the current situation onto the one considered previously,

$$\begin{aligned} \sigma = \left( \begin{array}{cccccc} {\overline{a}}_1 & \cdots & {\overline{a}}_{N-k} & a_1 & \cdots & a_k \\ 1 & \cdots & N-k & N-k+1 & \cdots & N \end{array} \right) \,. \end{aligned}$$

We can now make use of the preparation we did above and apply Eqn. 15 to see that,

22 $$\begin{aligned} \left( \mathcal {L}(G)_{[{\overline{A}},{\overline{A}}]} \right) _{i,j} = \left( \mathcal {L}(G^\sigma )_{[\sigma ({\overline{A}}), \sigma ({\overline{A}})]} \right) _{\tau _{\sigma , {\overline{A}}}(i), \tau _{\sigma , {\overline{A}}}(j)}\,, \end{aligned}$$

where we have used the permutation defined in Eqn. 14 for ${\overline{A}}$,

$$\begin{aligned} \tau _{\sigma , {\overline{A}}} = \theta _{\sigma ({\overline{A}})}^{-1} \left( \sigma \left( \theta _{{\overline{A}}} \right) \right) : \{1, \dotsc , N-k\} \rightarrow \{1, \dotsc , N-k\} \,. \end{aligned}$$

Since $\sigma (A) = B$ and $\sigma ({\overline{A}}) = {\overline{B}} = \{1, \dotsc , N - k\}$, it follows that $\theta _{\sigma ({\overline{A}})}$ is just the identity on $\left\{ 1, \dotsc , N - k \right\}$. Furthermore, $\sigma ({\overline{a}}_i) = i$, which means that,

23 $$\begin{aligned} \begin{aligned} \theta _{{\overline{A}}}(i) = {\overline{a}}_i = \sigma ^{-1}(i) \qquad \text{ for }\,\, i = 1, \dotsc , N-k \,. \end{aligned} \end{aligned}$$

It follows that, on ${\overline{A}}$, $\sigma = \theta _{{\overline{A}}}^{-1}$. Putting these pieces together, it then follows from Eqn. 14 that $\tau _{\sigma , {\overline{A}}}$ is the identity on $\left\{ 1, \dotsc , N-k \right\}$. Hence, Eqn. 22 becomes,

$$\begin{aligned} \left( \mathcal {L}(G)_{[{\overline{A}},{\overline{A}}]} \right) _{i,j} = \left( \mathcal {L}(G^\sigma )_{[{\overline{B}}, {\overline{B}}]} \right) _{i,j} \,, \end{aligned}$$

so that we may, in fact, just equate these two matrices, $\mathcal {L}(G)_{[{\overline{A}},{\overline{A}}]} = \mathcal {L}(G^\sigma )_{[{\overline{B}}, {\overline{B}}]}$. Since $B = \left\{ N-k+1, \dotsc , N \right\}$, we can now appeal to the first part of the proof to deduce that,

$$\begin{aligned} \left( \mathcal {L}(G^\sigma )_{[{\overline{B}},{\overline{B}}]} \right) ^{-1}_{i,j} = -\frac{w(\Phi _{B \cup \{j\} \rightarrow B \cup \{i\}}(G^\sigma ))}{w(\Phi _B(G^\sigma ))} \,. \end{aligned}$$

It follows using Eqns. 16 and 23 that,

$$\begin{aligned} w(\Phi _{A \cup \{\theta _{{\overline{A}}}(j)\} \rightarrow A \cup \{\theta _{{\overline{A}}}(i)\}}(G))&= w(\Phi _{\sigma (A) \cup \{\sigma (\theta _{{\overline{A}}}(j))\} \rightarrow \sigma (A) \cup \{\sigma (\theta _{{\overline{A}}}(i))\}}(G^\sigma )) \\&= w(\Phi _{B \cup \{j\} \rightarrow B \cup \{i\}}(G^\sigma )) \,, \end{aligned}$$

and that,

$$\begin{aligned} w(\Phi _A(G)) = w(\Phi _{\sigma (A)}(G^\sigma )) = w(\Phi _B(G^\sigma )) \,. \end{aligned}$$

Putting all these pieces together, we finally obtain,

$$\begin{aligned} \left( \mathcal {L}(G)_{[{\overline{A}},{\overline{A}}]} \right) ^{-1}_{i,j}&= \left( \mathcal {L}(G^\sigma )_{[{\overline{B}},{\overline{B}}]} \right) ^{-1}_{i,j} \\&= -\frac{w(\Phi _{B \cup \{j\} \rightarrow B \cup \{i\}}(G^\sigma ))}{w(\Phi _B(G^\sigma ))} \\&= -\frac{w(\Phi _{A \cup \{\theta _{{\overline{A}}}(j)\} \rightarrow A \cup \{\theta _{{\overline{A}}}(i)\}}(G))}{w(\Phi _A(G))} \,, \end{aligned}$$

as required. $$\square $$

The following elementary result on spanning forests will also be helpful in what follows. Choose $A \subseteq \mathcal {V}(G)$ and $i, j \in {\overline{A}}$ such that the set, $\Phi _{A \cup \{i\} \rightarrow A \cup \{j\}}(G)$, of spanning forests rooted at $A \cup \{j\}$ with a path from *i* to *j* is non-empty, and let *F* be such a spanning forest. If *G* contains an edge $j \rightarrow z$ for some other root $z \in A$, then adjoining this edge to *F* yields a spanning forest that is rooted at *A* and contains a path from *i* to *z*. That is, the new spanning forest lies in $\Phi _{(A \setminus \{z\}) \cup \{i\} \rightarrow A}(G)$. Conversely, if $F \in \Phi _{(A \setminus \{z\}) \cup \{i\} \rightarrow A}(G)$, then *F* must contain a path from *i* to *z*. Since *i* is itself not a root, this path must possess at least one edge; if the last edge on this path is $j \rightarrow z$, then $j \not \in A$ and omitting this edge creates a forest in $\Phi _{A \cup \{i\} \rightarrow A \cup \{j\}}(G)$. These procedures are evidently inverse to each other.

##### Lemma 3

Given $A \subseteq \mathcal {V}(G)$, $i \in {\overline{A}}$ and $z \in A$, the map,

$$\begin{aligned} \bigcup _{j \in \textrm{V}_{\text {in}}(z) \setminus A}{\Phi _{A \cup \{i\} \rightarrow A \cup \{j\}}(G)} \rightarrow \Phi _{(A \setminus \{z\}) \cup \{i\} \rightarrow A}(G) \,, \end{aligned}$$

that adjoins the edge $j \rightarrow z$ is a bijection.

It follows immediately from Lemma 3 that, under the same assumptions,

24 $$\begin{aligned} \sum _{j \in \textrm{V}_{\text {in}}(z) \setminus A}w(\Phi _{A \cup \{i\} \rightarrow A \cup \{j\}}(G)) \, \ell (j \rightarrow z) = w(\Phi _{(A \setminus \{z\}) \cup \{i\} \rightarrow A}(G)) \,. \end{aligned}$$

### FPTs in graphs with singleton terminal SCCs

With the above preliminaries in hand, we can embark on calculating FPTs. This will require several steps. We will derive the basic time-evolution equation, use the Laplace transform to calculate moment-like quantities and then obtain formulas for the splitting probabilities and the moments of the conditional FPT distribution.

#### Definitions and notation

To simplify the argument, we will assume that each terminal SCC is a singleton, which we will refer to as a *terminal vertex*. Let *Z* denote the set of terminal vertices. More general situations can be reduced to this special case, as we will explain subsequently.

Let $X(\cdot )$ be the Markov process associated with *G*, and choose some starting vertex $i \in \mathcal {V}(G)$ and some terminal vertex $z \in Z$. We will argue somewhat intuitively here, to avoid getting into measure-theoretic complexities; see Anderson (1991); Norris (1997); Serfozo (2009) for the rigorous underpinnings. We consider stochastically generating a large finite ensemble, *E*, of unbounded trajectories of $X(\cdot )$ each starting from *i*, so that $X(0) = i$. With probability one, each trajectory will reach some terminal vertex in *Z* and stay there from then on. Let $A_{z,t} \subseteq E$ be the subset of those trajectories that have reached *z* by time *t*. The probability of reaching *z* from *i* by time *t* is then given by,

25 $$\begin{aligned} \begin{aligned} p_{i,z}(t) = \Pr \left[ X(t') = z\,\, \text{ for } \text{ some } \,\,0 \le t' \le t \mid X(0) = i\right] \approx \frac{\# A_{z,t}}{\# E} \,, \end{aligned} \end{aligned}$$

where the approximation becomes exact in the limit of an infinite ensemble. Since $A_{z,t}$ clearly enlarges with *t*, $p_{i,z}(t)$ must be non-decreasing, $p_{i,z}(t_1) \le p_{i,z}(t_2)$ if $t_1 \le t_2$, so that it resembles a cumulative distribution function. However, it is not a valid cumulative distribution function over *t* because it is not correctly normalised: the quantity $\lim _{t \rightarrow \infty }p_{i,z}(t)$, which we denote for convenience as $p_{i,z}(\infty )$, is not 1 but, rather, the *splitting probability*, $\pi _{i,z}$, of eventually reaching *z* from *i*. Let $B_z \subseteq E$ be the subset of trajectories that eventually reach *z*. Then,

26 $$\begin{aligned} \begin{aligned} \pi _{i,z} = \Pr \left[ X(t) = z \,\,\text{ for } \text{ some }\,\, t \ge 0 \mid X(0) = i \right] \approx \frac{\# B_z}{\# E} \,. \end{aligned} \end{aligned}$$

For sufficiently large *t*, $A_{z,t} = B_z$, so it follows from Eqn. 25 that $p_{i,z}(\infty ) = \pi _{i,z}$. This splitting probability is one of the quantities that we want to calculate.

The other quantities of interest arise from the conditional FPT distribution, which we can approach through the probability, $p_{c,i,z}(t)$, of reaching *z* from *i* by time *t*, conditioned on reaching *z* eventually. For this probability to be well-defined, it is necessary that $i \in \mathcal {V}_{\text {in}} (z)$, so that $B_z \not = \varnothing$. With that proviso,

27 $$\begin{aligned} \begin{aligned} p_{c,i,z}(t)&= \Pr [ X(t') = z \,\,\text{ for } \text{ some }\,\, 0 \le t' \le t \,\,\mid \\ &\qquad \qquad X(0) = i \,\,\text{ and }\,\, X(u) = z\,\, \text{ for } \text{ some }\,\, u > 0] \\ &\approx \frac{\# A_{z,t}}{\# B_z} \,. \end{aligned} \end{aligned}$$

It follows from Eqns. 26 and 27 that, as long as $i \in \mathcal {V}_{\text {in}} (z)$,

28 $$\begin{aligned} p_{c,i,z}(t) = \frac{p_{i,z}(t)}{\pi _{i,z}} \,. \end{aligned}$$

In contrast to $p_{i,z}(t)$, $p_{c,i,z}(t)$ is a cumulative distribution function that is correctly normalised with respect to *t*, with $p_{c,i,z}(\infty ) = 1$. Its time derivative, $dp_{c,i,z}(t) / dt$, gives the probability density of the conditional FPT distribution over *t*. We want to calculate the moments of this distribution.

We also consider the probability to reach any terminal vertex in *Z*. Let $A_t \subseteq E$ be those trajectories that have reached *Z* by time *t*. Then we can define,

29 $$\begin{aligned} \begin{aligned} p_{i,Z}(t) = \Pr \left[ X(t') \in Z\,\, \text{ for } \text{ some }\,\, 0 \le t' \le t \mid X(0) = i\right] \approx \frac{\# A_t}{\# E} \,. \end{aligned} \end{aligned}$$

Evidently, $p_{i,Z}(\infty ) = 1$, so that $p_{i,Z}(t)$ is also a bona fide cumulative distribution function, whose corresponding probability density is given by $dp_{i,Z}(t) / dt$. It is also clear that $A_t = \bigcup _{z \in Z}A_{z,t}$ and that this union is disjoint, so that $\# A_t = \sum _{z \in Z} \# A_{z,t}$. It follows from Eqns. 26 and 27 that,

30 $$\begin{aligned} p_{i,Z}(t) = \sum _{z \in Z : i \in \mathcal {V}_{\text {in}} (z)} p_{c,i,z}(t) \, \pi _{i,z} \,. \end{aligned}$$

Instead of working directly with $p_{c,i,z}(t)$ to calculate FPTs, it is easier to work with $p_{i,z}(t)$, for which we can find a system of differential equations for $i \in \mathcal {V}_{\text {in}} (z)$. From there, via the Laplace transform, we can determine the splitting probabilities, $\pi _{i,z}$, the moments of the conditional FPT to each terminal vertex, and the moments of the FPT to any terminal vertex.

We need some further notation before embarking on this calculation. Let *G* be a graph with $T \ge 1$ terminal SCCs. We can assume, without loss of generality, that the vertices are indexed so that the terminal SCCs are as follows,

$$\begin{aligned} \mathcal {T}(G) = \left\{ \left\{ N-T+1 \right\} , \dotsc , \left\{ N \right\} \right\} \,, \end{aligned}$$

so that $Z = \left\{ N-T+1, \dotsc , N \right\}$. If $i \in \mathcal {V}(G)$, then it must have a path to some terminal vertex. Let $\mathcal {Z}(i) \subseteq Z$ denote the subset of those terminal vertices,

31 $$\begin{aligned} \mathcal {Z}(i) = \mathcal {V}_{\text {out}}(i) \cap Z \not = \varnothing \,. \end{aligned}$$

$\mathcal {Z}(i) = \{i\}$ if, and only if, *i* is itself terminal.

Let us now choose $z \in Z$. It will be held fixed for much of the argument that follows. The vertices that lead to *z*, excluding *z* itself, which form the subset $\mathcal {V}_{\text {in}} (z) \setminus \{z\}$, must all be non-terminal. We can, again without loss of generality, index these non-terminal vertices as $1, \dotsc , N_z$, for some $N_z \le N - T$. Note that this indexing depends on the choice of $z \in Z$. It follows that,

32 $$\begin{aligned} \mathcal {V}_{\text {in}} (z) = \left\{ 1, \dotsc , N_z, z \right\} . \end{aligned}$$

It will be helpful to let $\mathcal {V}_{\text {in}}^{-}(z)$ denote just the non-terminal vertices in $\mathcal {V}_{\text {in}} (z)$, $\mathcal {V}_{\text {in}}^{-}(z) = \mathcal {V}_{\text {in}} (z) \setminus \{z\}$. Let us also choose $i \in \mathcal {V}_{\text {in}} (z)$, so that $z \in \mathcal {Z}(i)$. The vertex *i* will also be held fixed for much of the following argument.

#### The basic time-evolution equation

With that preparation, we can now derive a system of differential equations for $p_{i,z}(t)$. First note that, because $X(\cdot )$ is time-homogeneous, if we shift the time axis by *h*, so that trajectories start at $X(h) = i$, then the probability of reaching *z* by time $t + h$ is also given by $p_{i,z}(t)$. Recall from the elementary properties of Markov processes that the probability that $X(\cdot )$ remains at *i* up to time *t* is given by $e^{-\lambda _i t}$, where $\lambda _i$ is the total exit rate from *i*,

$$\begin{aligned} \lambda _i = \sum _{j \in \textrm{V}_{\text {out}}(i)}\ell (i \rightarrow j) \,. \end{aligned}$$

The probability that $X(\cdot )$ has left *i* by time *t* is then $1 - e^{-\lambda _i t}$. If we choose $0< h < t$ sufficiently small, then the probability that $X(\cdot )$ has taken one transition from *i* by time *h* is $\lambda _i h$, to first order in *h*. The probability that it has taken *k* transitions by time *h* can then be no less than order *k* in *h*. Accordingly, to first order in *h*, we may assume that $X(\cdot )$ has taken no more than one transition from *i*. There are then two mutually exclusive possibilities. First, $X(\cdot )$ remains at *i* up to time *h*, with probability $e^{-\lambda _i h}$, and then reaches *z* within the remaining time from *h* to *t*. Because of the time-homogeneity mentioned above, the latter probability is just $p_{i,z}(t-h)$. Second, $X(\cdot )$ leaves *i* by time *h*, which it does with probability $1 - e^{-\lambda _i h}$, and takes the edge $i \rightarrow j$, which it does with probability $\ell (i \rightarrow j)/\lambda _i$, and then reaches *z* from *j* within the remaining time from *h* to *t*, which, as before, occurs with probability $p_{j,z}(t-h)$. Putting these parts together, we see that,

$$\begin{aligned} p_{i,z}(t) = e^{-\lambda _i h} \, p_{i,z}(t-h) + (1 - e^{-\lambda _i h}) \sum _{j \in \textrm{V}_{\text {out}}(i)}\left( \frac{\ell (i \rightarrow j)}{\lambda _i} \right) p_{j,z}(t - h) + \textrm{o}(h) \,, \end{aligned}$$

where the “little o” notation, $\textrm{o}(h)$, denotes some function *f*(*h*) which is of order higher than 1 in *h*, so that $\lim _{h \rightarrow 0}f(h)/h = 0$. If we subtract $p_{i,z}(t - h)$ from both sides, divide through by *h*, and let $h \rightarrow 0$, then, keeping in mind that $\lim _{h \rightarrow 0}(1 - e^{-\lambda _i h})/h = \lambda _i$, we obtain,

33 $$\begin{aligned} \frac{d}{dt} p_{i,z}(t) = \sum _{j \in \textrm{V}_{\text {out}}(i)}\ell (i \rightarrow j) \, p_{j,z}(t) - \lambda _i \, p_{i,z}(t) \,. \end{aligned}$$

We could use Eqn. 28 to rewrite Eqn. 33 as a system of equations for the conditional probabilities, $p_{c,i,z}(t)$, but this would also involve the splitting probabilities, $\pi _{i,z}$, which are themselves unknown. This illustrates why it is preferable to work with the system of equations for the unconditional probabilities, $p_{i,z}(t)$, in Eqn. 33.

We can rewrite Eqn. 33 in terms of the underlying graph *G* by defining the vector of probabilities to reach *z* by time *t*, $\textbf{p}_z(t) = (p_{1,z}(t), \dotsc , p_{N_z,z}(t))^\textrm{T}\in \mathbb {R}^{N_z}$, where the indices run over the non-terminal vertices of *G* that lead to *z*, i.e., $i \in \mathcal {V}_{\text {in}}^{-}(z)$. (Note that, if $i = z$, then $p_{i,z}(t) = p_{z,z}(t) = 1$ for all $t \ge 0$.) Now, let $\textbf{p}_z^+(t) \in \mathbb {R}^{N_z + 1}$ denote the augmented vector,

$$\begin{aligned} \textbf{p}_z^+(t) = (p_{1,z}(t), \dotsc , p_{N_z,z}(t), p_{z,z}(t))^\textrm{T}= (p_{1,z}(t), \dotsc , p_{N_z,z}(t), 1)^\textrm{T}. \end{aligned}$$

Then, rewriting Eqn. 33, we see that,

34 $$\begin{aligned} \frac{d}{dt} \textbf{p}_z(t) = \left( \mathcal {L}(G)^\textrm{T}\right) _{[\mathcal {V}_{\text {in}}^{-}(z), \mathcal {V}_{\text {in}} (z)]} \textbf{p}_z^{+}(t) \,, \end{aligned}$$

where the submatrix of ${{\mathcal {L}}}(G)^\textrm{T}$ has size $N_z \times (N_z + 1)$. Eqn. 34 is the *adjoint master equation* of the Markov process (Iyer-Biswas et al. 2016; van Kampen 2007), so named because its operator is the transpose to that in the master equation (Eqn. 1).

#### The Laplace transform

The calculation of moments is most readily done by using the Laplace transform of the probability density. Let $u_{i,z}(t)$ be the time derivative of $p_{i,z}(t)$,

35 $$\begin{aligned} u_{i,z}(t) = \frac{dp_{i,z}(t)}{dt} \,. \end{aligned}$$

As noted above, $u_{i,z}(t)$ is generally not the density of a probability distribution over *t* because it is not correctly normalised, but it can serve the same purpose here. Differentiating Eqn. 34 with respect to *t* yields a similar adjoint time-evolution equation for $\textbf{u}_z(t) = \left( u_{1,z}(t), \dotsc , u_{N_z,z}(t) \right) ^\textrm{T}$,

36 $$\begin{aligned} \frac{d}{dt} \textbf{u}_z(t) = \left( \mathcal {L}(G)^\textrm{T}\right) _{[\mathcal {V}_{\text {in}}^{-}(z), \mathcal {V}_{\text {in}}^{-}(z)]} \textbf{u}_z(t) \,, \end{aligned}$$

where we have used the fact that $u_{z,z}(t) = 0$ for all $t \ge 0$ to drop the final column in the right-hand matrix, which now has size $N_z \times N_z$.

Now, let $\widetilde{u}_{i,z}(s) = \mathscr {L}{\left\{ u_{i,z}(t) \right\} }$ denote the Laplace transform of $u_{i,z}(t)$,

$$\begin{aligned} \widetilde{u}_{i,z}(s) = \mathscr {L}{\left\{ u_{i,z}(t) \right\} } = \int _0^\infty {e^{-st} \, u_{i,z}(t) \, dt} \,. \end{aligned}$$

Applying the Laplace transform to both sides of Eqn. 36 and using the well-known property that it converts differentiation by *t* into multiplication by *s*, we get a linear algebraic equation for $\widetilde{\textbf{u}}_z(s) = \left( \widetilde{u}_{1,z}(s), \dotsc , \widetilde{u}_{N_z,z}(s) \right) ^\textrm{T}$,

37 $$\begin{aligned} s \widetilde{\textbf{u}}_z(s) - \textbf{u}_z(0) = \left( \mathcal {L}(G)^\textrm{T}\right) _{[\mathcal {V}_{\text {in}}^{-}(z), \mathcal {V}_{\text {in}}^{-}(z)]} \widetilde{\textbf{u}}_z(s) \,. \end{aligned}$$

To determine the initial condition vector, $\textbf{u}_z(0)$, note that $p_{i,z}(0) = 0$ if $i \not = z$, and $p_{z,z}(0) = 1$. Substituting into Eqn. 33 for $t = 0$, we obtain,

38 $$\begin{aligned} u_{i,z}(0) = \left. \frac{dp_{i,z}(t)}{dt} \right| _{t = 0} = {\left\{ \begin{array}{ll} \ell (i \rightarrow z) & \text {if}\, i \rightarrow z \\ 0 & \text {otherwise.} \end{array}\right. } \end{aligned}$$

It follows that $\textbf{u}_z(0)$ contains those entries in the *z*-th column of $\mathcal {L}(G)^\textrm{T}$ that correspond to the vertices in $\mathcal {V}_{\text {in}}^{-}(z)$, which we will denote by $\textbf{v}$,

39 $$\begin{aligned} \textbf{v}= \textbf{u}_z(0) = \left( \mathcal {L}(G)^\textrm{T}\right) _{[\mathcal {V}_{\text {in}}^{-}(z), \{z\}]} \,. \end{aligned}$$

Therefore, we can now rewrite Eqn. 37 as,

40 $$\begin{aligned} \widetilde{\textbf{u}}_z(s) = \left( s \textbf{I}- \left( \mathcal {L}(G)^\textrm{T}\right) _{[\mathcal {V}_{\text {in}}^{-}(z), \mathcal {V}_{\text {in}}^{-}(z)]} \right) ^{-1} \textbf{v}\,. \end{aligned}$$

Proposition 2 tells us that the inverse of $\mathcal {L}(G)_{[\mathcal {V}_{\text {in}}^{-}(z), \mathcal {V}_{\text {in}}^{-}(z)]}$ exists if, and only if, there exists a spanning forest of *G* rooted at $\overline{\mathcal {V}_{\text {in}}^{-}(z)}$. Since $\overline{\mathcal {V}_{\text {in}}^{-}(z)}$ contains all the terminal vertices, *Z*, and every vertex in *G* leads to some terminal vertex, such a spanning forest must exist (§2.1.1). Because the determinant is a continuous function of the entries of a matrix, Eqn. 40 is well-defined within some neighbourhood of $s = 0$.

For notational convenience in what follows, we introduce the negative transpose of the Laplacian matrix, as

41 $$\begin{aligned} \textbf{L}(G) = -\mathcal {L}(G)^\textrm{T}\,, \end{aligned}$$

so that we can rewrite Eqn. 40 as,

42 $$\begin{aligned} \widetilde{\textbf{u}}_z(s) = \left( s \textbf{I}+ \textbf{L}(G)_{[\mathcal {V}_{\text {in}}^{-}(z), \mathcal {V}_{\text {in}}^{-}(z)]} \right) ^{-1} \textbf{v}\,. \end{aligned}$$

#### Calculating moments

With the Laplace transform $\widetilde{\textbf{u}}_z(s)$ at our disposal, we can now start to calculate moments. Although $u_{i,z}(t)$ is not a bona fide probability density over *t*, as noted above, we can still define the quantity corresponding to its *r*-th moment,

43 $$\begin{aligned} \mu _{i,z}^{(r)} = \int _0^\infty {t^r u_{i,z}(t) \, dt} = \left. \left( -1 \right) ^r \frac{d^r \widetilde{u}_{i,z}(s)}{ds^r} \right| _{s=0}\,, \end{aligned}$$

where the second equality comes from the well-known property of the Laplace transform that differentiation by *s* corresponds to multiplication by *t*. Introducing the vector $\varvec{\mu }^{(r)}_z = (\mu ^{(r)}_{1,z}, \dotsc , \mu ^{(r)}_{N_z,z})^{\textrm{T}}$ and using Eqn. 42, we can write,

$$\begin{aligned} \varvec{\mu }^{(r)}_z = (-1)^r \left. \frac{d^r}{ds^r} \left( s \textbf{I}+ \textbf{L}(G)_{[\mathcal {V}_{\text {in}}^{-}(z), \mathcal {V}_{\text {in}}^{-}(z)]} \right) ^{-1} \right| _{s=0} \textbf{v}\,. \end{aligned}$$

Recall that, if $\textbf{M}$ is a square matrix that does not depend on *s*, then (Bernstein 2009),

$$\begin{aligned} \frac{d^r}{ds^r} \left( s\textbf{I}+ \textbf{M} \right) ^{-1} = (-1)^r \, r! \left( s\textbf{I}+ \textbf{M} \right) ^{-(r+1)} \,. \end{aligned}$$

It follows that,

44 $$\begin{aligned} \varvec{\mu }^{(r)}_z = r! \left( \textbf{L}(G)_{[\mathcal {V}_{\text {in}}^{-}(z), \mathcal {V}_{\text {in}}^{-}(z)]} \right) ^{-(r+1)} \, \textbf{v}\,. \end{aligned}$$

Eqn. 44 immediately reveals how $\varvec{\mu }^{(r)}_z$ can be calculated by appealing to Proposition 2.

Before doing that, it will be helpful for what follows (see Theorem 6) to allow greater generality in the choice of the submatrix of $\textbf{L}(G)$ that we use. Suppose that we choose $U \subseteq \mathcal {V}(G)$ to be any subset of vertices that contains all the terminal vertices but does not contain any of the non-terminal vertices that lead to *z*,

45 $$\begin{aligned} \begin{aligned} Z \subseteq U&\subseteq \overline{\mathcal {V}_{\text {in}}^{-}(z)} \\ \mathcal {V}_{\text {in}}^{-}(z) \subseteq {\overline{U}}&\subseteq {\overline{Z}} \,. \end{aligned} \end{aligned}$$

Because *U* contains the terminal SCCs (in this case, terminal vertices) of *G*, it follows from what was said in §2.1.1 that $\Phi _U(G) \not = \varnothing$. Hence, Proposition 2 tells us that the submatrix $\textbf{L}(G)_{[{\overline{U}},{\overline{U}}]}$ is invertible. Moreover, suppose that $i > N_z$ and $1 \le j \le N_z$, so that $j \in \mathcal {V}_{\text {in}}^{-}(z)$. Then there can be no edge $i \rightarrow j$: if there were, then, because of our choice of indices, $i \in \mathcal {V}_{\text {in}} (z) = \{1, \dotsc , N_z, z\}$. But, since $i > N_z$, this means that $i = z$, which is terminal, so there can be no edge $z \rightarrow j$, which yields a contradiction. It follows that this submatrix has a block upper-triangular form,

46 $$\begin{aligned} \textbf{L}(G)_{[{\overline{U}}, {\overline{U}}]} = \left[ \begin{array}{cc} \textbf{A}& \textbf{B}\\ \textbf{0} & \textbf{C}\end{array} \right] \,, \end{aligned}$$

where $\textbf{A}= \textbf{L}(G)_{[\mathcal {V}_{\text {in}}^{-}(z), \mathcal {V}_{\text {in}}^{-}(z)]}$. Since we know that the block matrix in Eqn. 46 is invertible, we can apply the block matrix inversion formula (Bernstein 2009) to obtain,

$$\begin{aligned} \left( \textbf{L}(G)_{[{\overline{U}},{\overline{U}}]} \right) ^{-1} = \left[ \begin{array}{cc} \textbf{A}^{-1} & -\textbf{A}^{-1} \, \textbf{B}\, (\textbf{C}^{-1}) \\ \textbf{0} & \textbf{C}^{-1} \end{array} \right] \,. \end{aligned}$$

It follows that, provided $i, j \in \mathcal {V}_{\text {in}}^{-}(z)$,

$$\begin{aligned} \left( \textbf{L}(G)_{[{\overline{U}},{\overline{U}}]} \right) ^{-1}_{i,j} = \textbf{A}^{-1}_{i,j} = \left( \textbf{L}(G)_{[\mathcal {V}_{\text {in}}^{-}(z), \mathcal {V}_{\text {in}}^{-}(z)]} \right) ^{-1}_{i,j} \,. \end{aligned}$$

If we consider Proposition 2 with $A = U$, then, in view of Eqn. 45, $\{1, \dotsc , N_z\} \subseteq {\overline{U}}$, so that the ordering map, $\theta _{{\overline{U}}}: \{1, \dotsc , \# {\overline{U}}\} \rightarrow {\overline{U}}$, is the identity, $\theta _{{\overline{U}}}(q) = q$, so long as $q \in \mathcal {V}_{\text {in}}^{-}(z) = \{1, \dotsc , N_z\}$. Accordingly, we can apply Proposition 2 to conclude that,

47 $$\begin{aligned} \left( \textbf{L}(G)_{[\mathcal {V}_{\text {in}}^{-}(z), \mathcal {V}_{\text {in}}^{-}(z)]} \right) ^{-1}_{i,j} = \frac{w(\Phi _{U \cup \{i\} \rightarrow U \cup \{j\}}(G))}{w(\Phi _U(G))} \,, \end{aligned}$$

where $i, j \in \mathcal {V}_{\text {in}}^{-}(z)$ and *U* is chosen as in Eqn. 45.

#### Splitting probabilities and conditional FPT moments

We can now calculate the quantities of interest. First, if we set $r = 0$ in Eqn. 43 and recall Eqn. 35, then, for all $i \in \mathcal {V}_{\text {in}}^{-}(z)$, we have,

48 $$\begin{aligned} \mu _{i,z}^{(0)} = \int _{0}^\infty {u_{i,z}(t) \, dt} = p_{i,z}(\infty ) - p_{i,z}(0) = \pi _{i,z} \,. \end{aligned}$$

Therefore, we can use Eqn. 44 to write,

$$\begin{aligned} \pi _{i,z} = \sum _{j \in \mathcal {V}_{\text {in}}^{-}(z)}\left( \textbf{L}(G)_{[\mathcal {V}_{\text {in}}^{-}(z),\mathcal {V}_{\text {in}}^{-}(z)]} \right) _{i,j}^{-1} \, v_j \,. \end{aligned}$$

Making use of Eqns. 39 and 47, we then see that,

$$\begin{aligned} \pi _{i,z} = \sum _{j \in \textrm{V}_{\text {in}}(z)}{\left( \frac{w(\Phi _{U \cup \{i\} \rightarrow U \cup \{j\}}(G))}{w(\Phi _U(G))} \right) \ell (j \rightarrow z)} \,, \end{aligned}$$

where we recall that $\textrm{V}_{\text {in}}(z)$ is the subset of vertices with edges into *z*. We can now apply Eqn. 24, with $A = U$, to see that,

$$\begin{aligned} \pi _{i,z} = \frac{w(\Phi _{(U \setminus \{z\}) \cup \{i\} \rightarrow U}(G))}{w(\Phi _U(G))} \,. \end{aligned}$$

This leads to the first of our main results.

##### Theorem 4

Let *G* be a graph whose terminal SCCs are single vertices and let *Z* be the set of terminal vertices. Choose $z \in Z$. Then, for any subset $U \subseteq \mathcal {V}(G)$ such that $Z \subseteq U \subseteq \overline{\mathcal {V}_{\text {in}}^{-}(z)}$ and any $i \in \mathcal {V}_{\text {in}}^{-}(z)$, the splitting probability from *i* to *z* is given by,

49 $$\begin{aligned} \pi _{i,z} = \frac{w(\Phi _{(U \setminus \{z\}) \cup \{i\} \rightarrow U}(G))}{w(\Phi _U(G))}. \end{aligned}$$

##### Proof

Here, $T = \# Z$. We are at liberty to re-index the vertices of *G* so that $Z = \{N-T+1, \dotsc , N\}$ and, for the chosen $z \in Z$, $\mathcal {V}_{\text {in}} (z) = \{1, \dotsc , N_z, z\}$. The preceding calculation then yields Eqn. 49. $\square$

The denominator in Eqn. 49 is the weight of all spanning forests of *G* rooted at *U*, while the numerator is the weight of the subset of such spanning forests in which there is a path from *i* to *z*. Therefore, this ratio is a unitless number that must lie between 0 and 1, as expected for a probability. Moreover, since $i \in \mathcal {V}_{\text {in}}^{-}(z)$, there is at least one spanning forest that contributes to the numerator, which implies that $\pi _{i,z} \not = 0$.

Having calculated the splitting probability, we now turn to the moments of the conditional FPT distribution, which we denote by $\mu ^{(r)}_{c,i,z}$. It follows from Eqn. 28 that,

50 $$\begin{aligned} \mu _{c,i,z}^{(r)} = \frac{\mu _{i,z}^{(r)}}{\pi _{i,z}} \,. \end{aligned}$$

In view of Eqn. 48, we see that $\mu ^{(0)}_{c,i,z} = 1$. For the higher moments, we see from Eqn. 44 that,

$$\begin{aligned} \varvec{\mu }^{(r)}_z = r! \left( \textbf{L}(G)_{[\mathcal {V}_{\text {in}}^{-}(z), \mathcal {V}_{\text {in}}^{-}(z)]} \right) ^{-r} \varvec{\mu }^{(0)}_z \,, \end{aligned}$$

where Eqn. 48 tells us that $\varvec{\mu }^{(0)}_z = (\pi _{1,z}, \dotsc , \pi _{N_z,z})^{\textrm{T}}$. Expanding out the matrix multiplications, we see that,

$$\begin{aligned} \mu _{i,z}^{(r)} = r! \sum _{(i_1, \dotsc , i_r)} \left( \prod _{j=0}^{r-1}{\left( \textbf{L}(G)_{[\mathcal {V}_{\text {in}}^{-}(z), \mathcal {V}_{\text {in}}^{-}(z)]} \right) _{i_j,i_{j+1}}^{-1}} \right) \pi _{i_r,z} \,, \end{aligned}$$

where we have set $i_0 = i$ and the sum runs over $(i_1, \dotsc , i_r) \in (\mathcal {V}_{\text {in}}^{-}(z))^r$. It then follows from Eqn. 50 that,

51 $$\begin{aligned} \mu _{c,i,z}^{(r)} = \frac{r!}{\pi _{i,z}} \sum _{(i_1, \dotsc , i_r)} \left( \prod _{j=0}^{r-1}{\left( \textbf{L}(G)_{[\mathcal {V}_{\text {in}}^{-}(z), \mathcal {V}_{\text {in}}^{-}(z)]} \right) _{i_j,i_{j+1}}^{-1}} \right) \pi _{i_r,z} \,. \end{aligned}$$

Note that this equation is valid even when $r = 0$. In this case, the sum runs over the empty Cartesian product, $(\mathcal {V}_{\text {in}}^{-}(z))^0$, and the term corresponding to the sole 0-tuple contains an empty product, which evaluates to one. Furthermore, $\pi _{i_r,z} = \pi _{i_0,z} = \pi _{i,z}$. Therefore, we obtain $\mu _{c,i,z}^{(0)} = 1$, as before.

We can now deduce our second main result. As with Theorem 4, it is convenient to state this in slightly greater generality, in terms of a subset *U* that satisfies Eqn. 45.

##### Theorem 5

Let *G* be a graph whose terminal SCCs are single vertices and let *Z* be the set of terminal vertices. Choose $z \in Z$. Then, for any subset $U \subseteq \mathcal {V}(G)$ such that $Z \subseteq U \subseteq \overline{\mathcal {V}_{\text {in}}^{-}(z)}$ and $i \in \mathcal {V}_{\text {in}}^{-}(z)$, the *r*-th moment of the conditional FPT from *i* to *z* is given by

$$\begin{aligned} \mu ^{(r)}_{c,i,z} = r! \sum _{(i_1, \dotsc , i_r) \in {\overline{U}}^r}{\left( \prod _{j=0}^{r-1}{\frac{w(\Phi _{U \cup \{i_j\} \rightarrow U \cup \{i_{j+1}\}}(G))}{w(\Phi _U(G))}} \right) \left( \frac{w(\Phi _{(U \setminus \{z\}) \cup \{i_r\} \rightarrow U}(G))}{w(\Phi _{(U \setminus \{z\}) \cup \{i\} \rightarrow U}(G))} \right) } \,, \end{aligned}$$

where $$i_0 = i$$.

##### Proof

We undertake a similar re-indexing as in the proof of Theorem 4 and make use of the calculations above. We can rewrite the quantity in Eqn. 51 by using Eqn. 47 to get,

$$\begin{aligned} \mu ^{(r)}_{c,i,z} = r! \sum _{(i_1, \dotsc , i_r)}\left( \prod _{j=0}^{r-1}{\frac{w(\Phi _{U \cup \{i_j\} \rightarrow U \cup \{i_{j+1}\}}(G))}{w(\Phi _U(G))}} \right) \left( \frac{\pi _{i_r,z}}{\pi _{i,z}} \right) \,, \end{aligned}$$

where $(i_1, \dotsc , i_r) \in (\mathcal {V}_{\text {in}}^{-}(z))^r$. We can then use Theorem 4 to rewrite the ratio of splitting probabilities to get,

$$\begin{aligned} \mu ^{(r)}_{c,i,z} = r! \sum _{(i_1, \dotsc , i_r)}\left( \prod _{j=0}^{r-1}{\frac{w(\Phi _{U \cup \{i_j\} \rightarrow U \cup \{i_{j+1}\}}(G))}{w(\Phi _U(G))}} \right) \left( \frac{w(\Phi _{(U \setminus \{z\}) \cup \{i_r\} \rightarrow U}(G))}{w(\Phi _{(U \setminus \{z\}) \cup \{i\} \rightarrow U}(G))} \right) \,. \end{aligned}$$

This is the quantity in the statement of the Theorem, except that the summation is taken over $(i_1, \dotsc , i_r) \in (\mathcal {V}_{\text {in}}^{-}(z))^r$. To see that we can convert the sum to one that runs over ${\overline{U}}^r$, note that the *r*-fold product in the numerator of the first parenthetical term,

$$\begin{aligned} \prod _{j=0}^{r-1}{w(\Phi _{U \cup \{i_j\} \rightarrow U \cup \{i_{j+1}\}}(G))}, \end{aligned}$$

is nonzero if, and only if, there are paths

$$\begin{aligned} i_0 = i \leadsto i_1, \qquad i_1 \leadsto i_2, \qquad \dotsc , \qquad i_{r-1} \leadsto i_r \,. \end{aligned}$$

Furthermore, the weight in the numerator of the second parenthetical term is nonzero if, and only if, $i_r \leadsto z$. Therefore, the summand corresponding to $(i_1, \dotsc , i_r)$ is zero if at least one vertex in the *r*-tuple does not have a path to *z*. In view of Eqn. 45, any elements in ${\overline{U}}$ that are not in $\mathcal {V}_{\text {in}}^{-}(z)$ do not contribute to the sum. Hence, we are at liberty to take $(i_1, \dotsc , i_r) \in {\overline{U}}^r$, as stated. $\square$

The *r*-th moment of the conditional FPT, $\mu ^{(r)}_{c,i,z}$, as given by the formula in Theorem 5, has units of (time)$^r$. It is instructive to see how this arises on the right-hand side of the formula. Recall that a spanning forest *F* has $N - \#\mathcal {R}(F)$ edges. Hence, the second parenthetical term in the right-hand sum is unitless. In the first term, the weight in the numerator incurs one fewer edge label, and corresponding unit of (time)$^{-1}$, than the weight in the denominator, so that the ratio has units of time. It follows that the right-hand side as a whole has units of (time)$^r$, as required.

We also note that the formula for $\mu ^{(r)}_{c,i,z}$ in Theorem 5 shares with that for the steady-state probabilities in Eqn. 5 the property of being a manifestly positive rational algebraic function of the edge labels.

As the mean is often of interest, it may be helpful to extricate this as a separate result. Under the same conditions as Theorem 5,

52 $$\begin{aligned} \mu _{c,i,z}^{(1)} = \sum _{j \in {\overline{U}}}{\left( \frac{w(\Phi _{U \cup \{i\} \rightarrow U \cup \{j\}}(G))}{w(\Phi _U(G))} \right) \left( \frac{w(\Phi _{(U \setminus \{z\}) \cup \{j\} \rightarrow U}(G))}{w(\Phi _{(U \setminus \{z\}) \cup \{i\} \rightarrow U}(G))} \right) } \,. \end{aligned}$$

#### The unconditional FPT to *Z*

It follows readily from Eqn. 30 that the *r*-th moment of the FPT distribution to reach any of the terminal vertices in *Z* from *i* is given by,

53 $$\begin{aligned} \mu _{i,Z}^{(r)} = \sum _{z \in \mathcal {Z}(i)} \mu _{c,i,z}^{(r)} \, \pi _{i,z} \,, \end{aligned}$$

where we have made use of $\mathcal {Z}(i) \subseteq Z$, as introduced in Eqn. 31. Eqn. 53 allows us to deduce our third main result.

##### Theorem 6

Let *G* be a graph whose terminal SCCs are single vertices and let *Z* be the set of terminal vertices. The *r*-th moment of the FPT from $i \in {\overline{Z}}$ to *Z* is given by

$$\begin{aligned} \mu _{i,Z}^{(r)} = r! \sum _{(i_1, \dotsc , i_r) \in {\overline{Z}}^r}{\left( \prod _{j=0}^{r-1}{\frac{w(\Phi _{Z \cup \{i_j\} \rightarrow Z \cup \{i_{j+1}\}}(G))}{w(\Phi _Z(G))}} \right) }, \end{aligned}$$

where $$i_0 = i$$.

##### Proof

Let us take $U = Z$ and apply Theorems 4 and 5 to Eqn. 53 to obtain the following formula for $\mu ^{(r)}_{i,Z}$,

$$\begin{aligned} r! \sum _{z \in \mathcal {Z}(i)}{\sum _{(i_1, \dotsc , i_r) \in {\overline{Z}}^r}{\left( \prod _{j=0}^{r-1}{\frac{w(\Phi _{Z \cup \{i_j\} \rightarrow Z \cup \{i_{j+1}\}}(G))}{w(\Phi _Z(G))}} \right) \left( \frac{w(\Phi _{(Z \setminus \{z\}) \cup \{i_r\} \rightarrow Z}(G))}{w(\Phi _Z(G))} \right) }} \,. \end{aligned}$$

The *r*-fold product within the inner sum does not depend on *z*, so we can rearrange the formula to give,

$$\begin{aligned} r! \sum _{(i_1, \dotsc , i_r) \in {\overline{Z}}^r} \left( \prod _{j=0}^{r-1}{\frac{w(\Phi _{Z \cup \{i_j\} \rightarrow Z \cup \{i_{j+1}\}}(G))}{w(\Phi _Z(G))}} \right) \left( \sum _{z \in \mathcal {Z}(i)}{\frac{w(\Phi _{(Z \setminus \{z\}) \cup \{i_r\} \rightarrow Z}(G))}{w(\Phi _Z(G))}} \right) \,. \end{aligned}$$

As in the proof of Theorem 5, the *r*-fold product of weights in the numerator of the first parenthetical term is nonzero if, and only if, there are paths

54 $$\begin{aligned} i_0 = i \leadsto i_1, \qquad i_1 \leadsto i_2, \qquad \dotsc , \qquad i_{r-1} \leadsto i_r \,. \end{aligned}$$

Furthermore, the numerator in the second parenthetical term is nonzero if, and only if, $i_r \leadsto z$, so that $z \in \mathcal {Z}(i_r)$. Because $\leadsto$ is transitive, Eqn. 54 tells us that $i \leadsto i_r$. If $z' \in \mathcal {Z}(i_r)$, so that $i_r \leadsto z'$, then it follows that $i \leadsto z'$, so that $z' \in \mathcal {Z}(i)$. Hence, $\mathcal {Z}(i_r) \subseteq \mathcal {Z}(i)$ for any *r*-tuple for which the corresponding summand above is nonzero. Therefore, we can rewrite the second parenthetical expression as,

$$\begin{aligned} \sum _{z \in \mathcal {Z}(i_r)} \frac{w(\Phi _{(Z \setminus \{z\}) \cup \{i_r\} \rightarrow Z}(G))}{w(\Phi _Z(G))} \,. \end{aligned}$$

But now we see from Theorem 4 that this expression is nothing other than the total sum of splitting probabilities from $i_r$,

$$\begin{aligned} \sum _{z \in \mathcal {Z}(i_r)} \pi _{i_r,z} = 1 \,, \end{aligned}$$

from which the result follows. $\square$

In a similar fashion to the formula in Theorem 5, the formula in Theorem 6 has the required units of (time)$^{r}$ and is a manifestly positive rational algebraic function of the edge labels.

As before, it may be helpful to extricate from Theorem 6 the mean FPT from *i* to *Z*, which is given by,

55 $$\begin{aligned} \mu _{i,Z}^{(1)} = \sum _{j \in {\overline{Z}}}{\frac{w(\Phi _{Z \cup \{i\} \rightarrow Z \cup \{j\}}(G))}{w(\Phi _Z(G))}}. \end{aligned}$$

#### A single terminal vertex

The case of a single terminal vertex, *z*, is often encountered and it may be helpful to see what the results above yield in this case. Evidently, $\mathcal {V}_{\text {in}} (z) = \mathcal {V}(G)$. Taking $U = \{z\}$, as we would expect, Theorem 4 implies that the splitting probability from every vertex is one,

56 $$\begin{aligned} \pi _{i,z} = \frac{w(\Phi _{\{i\} \rightarrow \{z\}}(G))}{w(\Phi _{\{z\}}(G))} = 1 \,, \end{aligned}$$

since every spanning tree in $\Phi _{\{z\}}(G)$ has a path from *i* to *z*. It is also clear that

$$\begin{aligned} \mu _{c,i,z}^{(r)} = \mu _{i,z}^{(r)} = \mu ^{(r)}_{i,\{z\}} \,, \end{aligned}$$

where the first equality follows from Eqn. 50 and the second from Eqn. 53. We can summarise this as follows.

##### Proposition 7

Let *G* be a graph with one terminal vertex, *z*. Then the *r*-th moment of the FPT from $i \in \mathcal {V}(G)$ to *z* is given by

$$\begin{aligned} \mu _{i,z}^{(r)} = r! \sum _{(i_1, \dotsc , i_r) \in \left( \mathcal {V}(G) \setminus \{z\} \right) ^r} \left( \prod _{j=0}^{r-1}{\frac{w(\Phi _{\{i_j,z\} \rightarrow \{i_{j+1},z\}}(G))}{w(\Phi _{\{z\}}(G))}} \right) \,, \end{aligned}$$

where $$i_0 = i$$.

As before, it may be helpful to extricate from Proposition 7 the result for the mean FPT from *i* to *z*,

57 $$\begin{aligned} \mu _{i,z}^{(1)} = \sum _{j \in \mathcal {V}(G) \setminus \{z\}}{\frac{w(\Phi _{\{i,z\} \rightarrow \{j,z\}}(G))}{w(\Phi _{\{z\}}(G))}}\,. \end{aligned}$$

### FPTs on general graphs

**Fig. 3:**
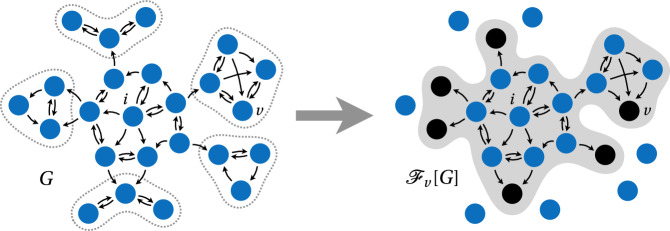
The edge removal procedure for FPT calculations on general graphs. The graph, *G*, on the left has five terminal SCCs, shown within dashed ovals, and two non-terminal SCCs. The source vertex *i* and the target vertex *v* are labelled. Note that *v* is in a terminal SCC. The edge removal procedure described in the text yields the graph, ${\mathcal {F}}_v[G]$, in the shaded region on the right. As implied by Lemma 8, the terminal SCCs of ${\mathcal {F}}_v[G]$ are all singletons, and are shown in black

Our main results above, Theorems 4, 5 and 6, were formulated for graphs whose terminal SCCs are singletons. Here, we show how these results can be extended to more general situations through a process of removing an appropriate set of edges.

Let *G* be a connected graph with any number of terminal SCCs, each of which may contain any number of vertices, and let $X(\cdot )$ be the associated Markov process. Let $v \in \mathcal {V}(G)$ be any vertex, which need not lie within a terminal SCC. We want to calculate the splitting probability and conditional FPT to *v* from some vertex, $i \in \mathcal {V}_{\text {in}} (v)$. To determine these quantities, first define a modified graph in which the outgoing edges from *v* are removed, along with the outgoing edges from any vertex in a terminal SCC, unless that SCC contains *v* itself. More precisely, the outgoing edges are removed from the set of vertices,

$$\begin{aligned} \begin{aligned} \left\{ v \right\} \cup \left( \bigcup _{C \in \mathcal {T}(G)\,,\, v \not \in C}{C} \right) \,. \end{aligned} \end{aligned}$$

This procedure is illustrated in Fig. 3. As shown in the figure, it may yield a graph with several connected components. Let ${\mathcal {F}}_v[G]$ denote the connected component of the modified graph that contains *v*, as shown with a shaded background in Fig. 3.

#### Lemma 8

The terminal SCCs of ${\mathcal {F}}_v[G]$ are all singletons. Furthermore, if $i \in \mathcal {V}_{\text {in}} (v)$, then the trajectory probabilities from *i* defined in Eqn. 25 are identical in *G* and in ${\mathcal {F}}_v[G]$, so that $p^G_{i,v}(t) = p^{{\mathcal {F}}_v[G]}_{i,v}(t)$.

#### Proof

The edge-removal procedure only removes edges, so that $\mathcal {V}({\mathcal {F}}_v[G]) \subseteq \mathcal {V}(G)$ and the SCCs of *G* can only break up into SCCs of ${\mathcal {F}}_v[G]$. If $C \in \mathcal {C}(G) \setminus \mathcal {T}(G)$ and $C \not \ni v$, then the procedure does nothing to it. Since *C* is non-terminal, it must have edges leaving it that are not removed by the procedure, so *C* remains a non-terminal SCC of ${\mathcal {F}}_v[G]$. If $C \in \mathcal {T}(G)$ and $C \not \ni v$, then the procedure disintegrates it so that only those vertices $j \in C$ with an incoming edge from outside *C* are retained in ${\mathcal {F}}_v[G]$. Each such *j* becomes a singleton terminal SCC, $\{j\} \in \mathcal {T}({\mathcal {F}}_v[G])$. Finally, if $C \ni v$, then the procedure removes the outgoing edges from *v* and thereby breaks up the strongly connected structure of *C* so that *v* becomes a terminal SCC, $\{v\} \in \mathcal {T}({\mathcal {F}}_v[G])$. Accordingly, the only terminal SCCs of ${\mathcal {F}}_v[G]$ are singletons.

For the second claim, we will argue as we did for Eqn. 25. Given $i \in \mathcal {V}_{\text {in}} (v)$, consider stochastically generating a large finite ensemble of unbounded trajectories of $X(\cdot )$ starting from *i*. With probability one, each of these trajectories will either reach *v* at some time or it will not. If a trajectory reaches *v* for the first time, truncate it at that time and extend it indefinitely while remaining at *v*. If a trajectory does not reach *v*, it will eventually reach a terminal SCC, $C \in \mathcal {T}(G)$, which does not contain *v*. In this case, truncate the trajectory at the first vertex $j \in C$ that is reached and extend it indefinitely while remaining at *j*. From the construction above, we see that $\{j\} \in \mathcal {T}({\mathcal {F}}_v[G])$. Each truncated trajectory is a trajectory of the Markov process associated with ${\mathcal {F}}_v[G]$ and this truncation process gives rise to a bona fide ensemble of trajectories in this latter Markov process. As the sizes of the two ensembles go to infinity, the probabilities of the original and truncated trajectories become identical, from which the second claim follows. $\square$

In view of Lemma 8, the splitting probabilities and the moments of the conditional FPT distribution from *i* to *v* in *G* can now be calculated by applying Theorems 4 and 5, respectively, to ${\mathcal {F}}_v[G]$.

## Examples

**Fig. 4:**
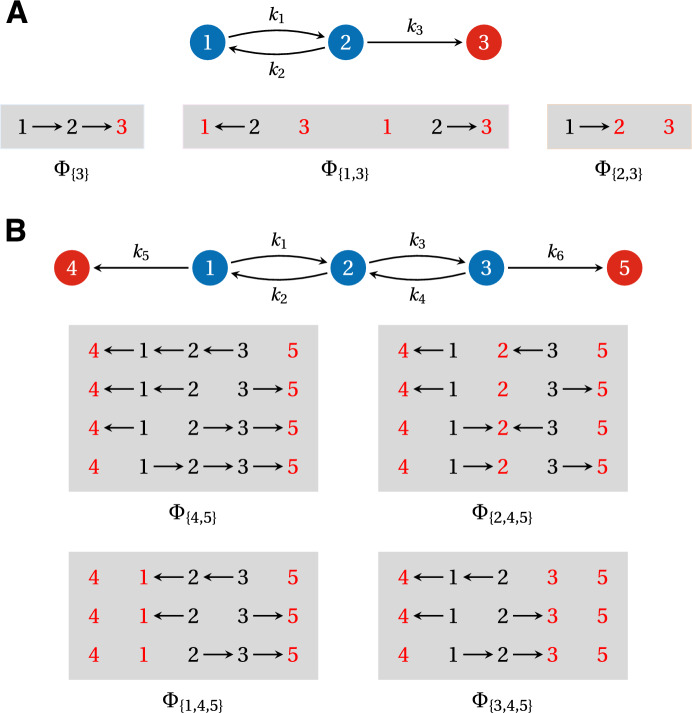
(**A**) A pipeline graph on three vertices, as discussed further in the text. The spanning forests are shown below. Red signifies a root vertex. (**B**) A pipeline graph on five vertices, as discussed further in the text, with the relevant spanning forests shown below. Red signifies a root vertex

**Fig. 5:**
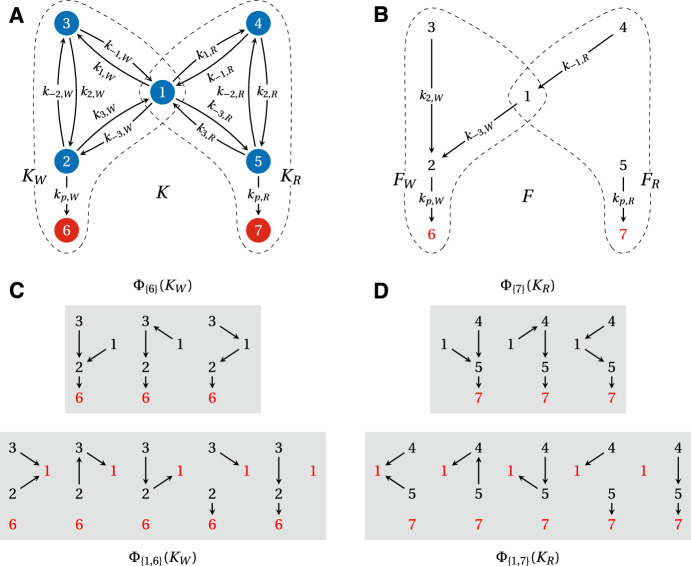
(**A**) A butterfly graph, *K*, adapted from Banerjee et al. (2017, Fig. 1C and D), as discussed further in the text. $K_R$ and $K_W$ denote the “right” and “wrong” subgraphs, respectively. (**B**) A spanning forest $F \in \Phi _{\{6,7\}}(K)$, showing how it splits into a spanning tree, $F_W$, of $K_W$ joined to a spanning forest, $F_R$, of $K_R$, as described in the text. (**C**, **D**) The spanning trees (top) and spanning forests (bottom) of $K_W$ rooted at 1 and 6 and of $K_R$ rooted at 1 and 7, respectively. Root vertices are in red font

In a prequel to the present paper, we summarised the main results discussed above without providing proofs and also worked through some example calculations for *pipeline graphs* (Nam and Gunawardena 2023, §2.4). These graphs consist of sequences of vertices with edges only between consecutive vertices (Fig. 4). The corresponding Markov processes have been widely studied in the context of single-molecule enzyme kinetics (Shaevitz et al. 2005; Kou et al. 2005; Chemla et al. 2008; Garai et al. 2009; Moffitt et al. 2010; Moffitt and Bustamante 2014). Pipeline graphs are particularly amenable to spanning forest enumeration, and we showed in Nam and Gunawardena (2023) how our results provide a systematic way to recover findings in the literature. Here, we will examine three examples, two pipeline graphs (Fig. 4) and a butterfly graph (Fig. 5), to illustrate in more detail how our formulas can be worked out in practice.

### Pipeline graphs

Consider first the pipeline graph in Fig. 4A, consisting of a pair of vertices linked by reversible edges, leading to a terminal vertex. This graph has been used to model a single catalytic cycle of a Michaelis–Menten enzyme mechanism (Shaevitz et al. 2005; Kou et al. 2005; Moffitt et al. 2010; Moffitt and Bustamante 2014), with the vertices representing enzyme states and the edges $1 \rightarrow 2$ corresponding to substrate binding, $2 \rightarrow 1$ to substrate unbinding and $2 \rightarrow 3$ to catalysis. The FPT from 1 to 3 is then a measure of the catalytic rate. Since there is only a single terminal vertex, we can use Proposition 7 to calculate the corresponding moments, $\mu ^{(r)}_{1,3}$, where

$$\begin{aligned} \begin{aligned} Z = \{ 3 \} \qquad \text{ and } \qquad \mathcal {V}_{\text{ in }}^{-} (3) = \{ 1, 2 \} \,. \end{aligned} \end{aligned}$$

We will omit the name of the graph in what follows, as it is clear from the context.

According to Proposition 7, we need to calculate a sum over the *r*-tuples,

$$\begin{aligned} (i_1, \dotsc , i_r) \in \left\{ 1,2 \right\} ^r \,, \end{aligned}$$

using spanning trees and forests drawn from the sets shown in Fig. 4A. There is only a single spanning tree rooted at 3, which contributes a weight of $k_1k_3$. As for the forests, the subsets required have the form $\Phi _{\{i_j,3\} \rightarrow \{i_{j+1},3\}}$, in which there is a path from $i_j$ to $i_{j+1}$ in the forest. Each such forest has only a single edge, and it follows from Fig. 4A that the forest weights are as given in the following table,

58 $$\begin{aligned} \begin{array}{|r|c|c|} \hline ~ & i_{j+1} = 1 & i_{j+1} = 2 \\ \hline i_j = 1 & k_2 + k_3 & k_1 \\ i_j = 2 & k_2 & k_1 \\ \hline \end{array} \,. \end{aligned}$$

The *r*-tuple, $(i_1, \dotsc , i_r)$, provides a code for reading off the corresponding product of forest weights, $w(\Phi _{\{i_0,3\} \rightarrow \{i_1,3\}}) \cdots w(\Phi _{\{i_{r-1},3\} \rightarrow \{i_{r},3\}})$, in the formula in Proposition 7.

For the mean FPT, we see from Eqn. 58 that,

59 $$\begin{aligned} \mu ^{(1)}_{1,3} = \sum _{i_1 \in \{1,2\}} \frac{w(\Phi _{\{1,3\} \rightarrow \{i_1,3\}})}{w(\Phi _{\{3\}})} = \frac{\left( k_2 + k_3 \right) + k_1}{k_1 k_3} \,. \end{aligned}$$

This formula is intuitively reasonable: if either $k_1$ or $k_3$ becomes comparatively large, the mean FPT approaches the reciprocal of the other rate; if $k_2$ becomes comparatively large, the mean FPT increases in proportion.

For the second moment, we see from Eqn. 58 that,

60 $$\begin{aligned} \begin{aligned} \mu ^{(2)}_{1,3}&= 2\sum _{(i_1,i_2) \in \{1,2\}^2}\left( \prod _{j = 0}^1 \frac{w(\Phi _{\{i_j,3\} \rightarrow \{i_{j+1},3\}})}{w(\Phi _{\{3\}})}\right) \\&= \frac{2 \left( \left( k_2+k_3 \right) ^2 + \left( k_2+k_3 \right) k_1 + k_1 k_2 + k_1^2 \right) }{\left( k_1 k_3 \right) ^2} \,. \end{aligned} \end{aligned}$$

The higher moments may be calculated in a similar way. It follows from Eqns. 59 and 60 that the quantity $\mu ^{(2)}_{1,3} - 2(\mu ^{(1)}_{1,3})^2$, which is similar to the variance, simplifies to $-2/(k_1 k_3)$. This quantity is independent of $k_2$, which may be helpful in experimentally testing whether an observed system can be reasonably described by the graph in Fig. 4A. It is plausible that similar polynomial simplifications exist between the higher moments, but this question lies outside the scope of the present paper.

We now consider the pipeline graph in Fig. 4B with five vertices, of which 4 and 5 are both terminal. Here,

$$\begin{aligned} Z = \{4, 5\} \quad \text {and} \quad {\mathcal {V}}_{\text {in}}^{-}(4)={\mathcal {V}}_{\text {in}}^{-}(5)= \{ 1, 2, 3 \} \,, \end{aligned}$$

and we will take the source vertex to be the central vertex 2. This graph was used by Terrell Hill to illustrate another method for calculating FPTs (Hill 1988), to which we will return in the Discussion, and we make use of a similar pipeline graph with 23 vertices in our analysis of the CRISPR–Cas9 system (Nam and Gunawardena 2025). Hill’s method permits calculation of the splitting probabilities, $\pi _{2,4}$ and $\pi _{2,5}$, and the mean unconditional FPT to any terminal vertex, $\mu ^{(1)}_{2,\{4,5\}}$, but not the mean conditional FPTs to specific terminal vertices, $\mu ^{(1)}_{c,2,4}$ and $\mu ^{(1)}_{c,2,5}$, which Hill calculated by “a separate stochastic argument (omitted).” In contrast, we can calculate all these quantities with our results, using the spanning forests shown in Fig. 4B.

Hill used a different notation, with the Markov transition rates given in the following table,61along with the symbols (Hill 1988, Eqn. 11),

62 $$\begin{aligned} \begin{aligned} A&= \beta + \alpha ' = k_4 + k_6 \\ B&= \delta + \gamma ' = k_1 + k_5 \\ \Sigma&= \gamma A + \alpha B + A B \\&= k_2 \left( k_4 + k_6 \right) + k_3 \left( k_1 + k_5 \right) + \left( k_4 + k_6 \right) \left( k_1 + k_5 \right) \,. \end{aligned} \end{aligned}$$

To calculate the splitting probabilities, we can use Theorem 4, with $U = Z = \{ 4, 5\}$, and the spanning forests in Fig. 4B, which give,

$$\begin{aligned} \pi _{2,4} = \frac{w(\Phi _{\{2,5\} \rightarrow \{4,5\}})}{w(\Phi _{\{4,5\}})} = \frac{k_2 k_5 \left( k_4 + k_6 \right) }{k_2 k_5 \left( k_4 + k_6 \right) + k_3 k_6 \left( k_1 + k_5 \right) } = \frac{\gamma \delta A}{\gamma \delta A + \alpha \beta B} \,, \end{aligned}$$

and similarly (or by noting that $\pi _{2,4} + \pi _{2,5} = 1$),

$$\begin{aligned} \pi _{2,5} = \frac{w(\Phi _{\{2,4\} \rightarrow \{4,5\}})}{w(\Phi _{\{4,5\}})} = \frac{k_3 k_6 \left( k_1 + k_5 \right) }{k_2 k_5 \left( k_4 + k_6 \right) + k_3 k_6 \left( k_1 + k_5 \right) } = \frac{\alpha \beta B}{\gamma \delta A + \alpha \beta B} \,. \end{aligned}$$

These formulas can be inferred from Hill (1988, Eqns. 12 and 13) by following the prescription given by Hill for determining the “fraction of walks that end with absorption” at a terminal vertex.

To calculate the mean unconditional FPT to *Z*, we can use the simplified version of Theorem 6 in Eqn. 55 with $Z = \{4,5\}$, which gives,

$$\begin{aligned} \mu ^{(1)}_{2,\{4,5\}} = \sum _{j \in \{1,2,3\}} \frac{w(\Phi _{\{2,4,5\} \rightarrow \{j,4,5\}})}{w(\Phi _{\{4,5\}})} \,. \end{aligned}$$

The spanning forests needed for this formula are shown in Fig. 4B. Recalling that $\Phi _{X \rightarrow X} = \Phi _X$, we see that,

63 $$\begin{aligned} \begin{aligned} w(\Phi _{\{2,4,5\} \rightarrow \{1,4,5\}})&= k_2 \left( k_4 + k_6 \right) \\ w(\Phi _{\{2,4,5\}})&= \left( k_1 + k_5 \right) \left( k_4 + k_6 \right) \\ w(\Phi _{\{2,4,5\} \rightarrow \{3,4,5\}})&= k_3 \left( k_1 + k_5 \right) \\ w(\Phi _{\{4,5\}})&= k_2 k_5 \left( k_4 + k_6 \right) + k_3 k_6 \left( k_1 + k_5 \right) \,. \end{aligned} \end{aligned}$$

It then follows that,

$$\begin{aligned} \mu ^{(1)}_{2,\{4,5\}} = \frac{k_2 \left( k_4 + k_6 \right) + \left( k_1 + k_5 \right) \left( k_4 + k_6 \right) + k_3 \left( k_1 + k_5 \right) }{k_2 k_5 \left( k_4 + k_6 \right) + k_3 k_6 \left( k_1 + k_5 \right) } = \frac{\Sigma }{\gamma \delta A + \alpha \beta B} \,. \end{aligned}$$

which recovers Hill (1988, Eqn. 13).

To calculate the mean conditional FPT from 2 to 4, we can use Theorem 5 with $U = Z = \{4,5\}$, which yields,

$$\begin{aligned} \mu ^{(1)}_{c,2,4} = \sum _{i_1 \in \{1,2,3\}} \left( \frac{w(\Phi _{\{2,4,5\} \rightarrow \{i_1,4,5\}})}{w(\Phi _{\{4,5\}})}\right) \left( \frac{w(\Phi _{\{i_1,5\} \rightarrow \{4,5\}})}{w(\Phi _{\{2,5\} \rightarrow \{4,5\}})} \right) \,. \end{aligned}$$

We need some more spanning forests to calculate this, in addition to those given in Eqn. 63. Examining Fig. 4B, we see that,

$$\begin{aligned} \begin{aligned} w(\Phi _{\{1,5\} \rightarrow \{4,5\}})&= k_5 \left( k_2 k_4 + k_2 k_6 + k_3 k_6 \right) \\ w(\Phi _{\{2,5\} \rightarrow \{4,5\}})&= k_2 k_5 \left( k_4 + k_6 \right) \\ w(\Phi _{\{3,5\} \rightarrow \{4,5\}})&= k_2 k_4 k_5 \,. \end{aligned} \end{aligned}$$

It follows that,

$$\begin{aligned} \mu _{c,2,4}^{(1)}&= \frac{\left( k_4 + k_6 \right) \left( k_2 k_4 + k_2 k_6 + k_3 k_6 \right) + \left( k_1 + k_5 \right) \left( k_4 + k_6 \right) ^2 + k_3 k_4 \left( k_1 + k_5 \right) }{\left( k_4 + k_6 \right) \left( k_2 k_5 \left( k_4 + k_6 \right) + k_3 k_6 \left( k_1 + k_5 \right) \right) } \\&= \frac{\gamma A^2 + \alpha \beta A + BA^2 + \alpha \alpha ' B}{A(\gamma \delta A + \alpha \beta B)} \,, \end{aligned}$$

which recovers the corresponding formula in Hill (1988, Eqn. 14). The formula for $\mu ^{(1)}_{c,2,5}$ may be calculated in a similar way. As noted above, Hill’s method, in contrast to ours, does not extend to these conditional FPTs, which he determined separately, nor to higher moments than the mean, which he did not calculate. We will provide such generalisations of Hill’s approach in a sequel to the present paper (Nam and Gunawardena 2025).

Pipeline graphs have relatively simple structures, which lend themselves to these calculations (Nam and Gunawardena 2023). However, enumerating spanning trees or forests rapidly becomes intractable as the graph becomes larger or more complex. Methods have emerged within the linear framework for dealing with this combinatorial explosion and they are explained further in Nam et al. (2022); Nam and Gunawardena (2023). It is also possible to exploit symmetries in the graph, as the next example shows.

### A butterfly graph

Fig. 5A shows a *butterfly graph*, consisting of two structurally identical “wings” that intersect at a single vertex. Butterfly graphs were defined in Wong et al. (2018) to formalise John Hopfield’s concept of discrimination by kinetic proofreading (Hopfield 1974). They have been widely used in studies of discrimination (Murugan et al. 2014; Banerjee et al. 2017; Mallory et al. 2019; Çetiner and Gunawardena 2022), although more often as a diagram than as a mathematical object, as we use them here. One wing of the butterfly graph describes a discriminatory mechanism when it is processing the “right” substrate, while the other wing describes the mechanism when it is processing the “wrong” substrate. The structural symmetry reflects the assumption that the mechanism behaves in an identical way with regard to either substrate except for the transition rates. The edge labels may therefore be different between the two wings.

The graph *K* in Fig. 5A is taken from the paper of Banerjee et al. (2017), where it was used to model the discriminatory process during stepwise incorporation of monomers in mRNA translation and in DNA replication. For convenience, we use the same notation for the edge labels in *K* as in Banerjee et al. (2017) but we have followed our integer convention for vertices in place of the names used in Banerjee et al. (2017).

Banerjee et al. (2017) considered the ratio of splitting probabilities,

$$\begin{aligned} \eta = \frac{\pi _{1,6}(K)}{\pi _{1,7}(K)}, \end{aligned}$$

as a measure of accuracy in translation or replication. Theorem 4 tells us that,

64 $$\begin{aligned} \eta = \frac{w(\Phi _{\{1,7\} \rightarrow \{6,7\}}(K))}{w(\Phi _{\{1,6\} \rightarrow \{6,7\}}(K))}. \end{aligned}$$

Enumerating the required spanning forests is considerably simplified by exploiting the symmetry of the butterfly graph. Let $K_R$ and $K_W$ be the subgraphs of *K* corresponding to the “right” and “wrong” wings of *K*, respectively (Fig. 5A). We can think of *K* as arising from the union of $K_R$ and $K_W$ by identifying them at the common vertex 1. We previously introduced the following notation (Wong et al. 2018),

$$\begin{aligned} K = K_W \oplus _1 K_R \,, \end{aligned}$$

for this joining operation on graphs, where the subscript on the operator $\oplus$ specifies the common identified vertex. Now consider a spanning forest, $F \in \Phi _{\{6,7\}}(K)$. If 1 leads to 6 in *F*, so that $F \in \Phi _{\{1,7\} \rightarrow \{6,7\}}(K)$, as shown in Fig. 5B, then the other vertices of *F* in $K_W$ must also lead to 6 in *F*. This means that the subgraph of *F* in $K_W$ is a spanning tree of $K_W$ rooted at 6, denoted $F_W \in \Phi _{\{6\}}(K_W)$. As for the vertices of *K* that lie in $K_R$, each such vertex must either lead to 6 or to 7 in *F*. If a vertex leads to 6, it must do so via 1. Therefore, the subgraph of *F* in $K_R$ must be a spanning forest rooted at 1 and 7, denoted $F_R \in \Phi _{\{1,7\}}(K_R)$. We see that,

$$\begin{aligned} F = F_W \oplus _1 F_R \,. \end{aligned}$$

A similar argument works when 1 leads to 7 in *F*, so that $F \in \Phi _{\{1,6\} \rightarrow \{6,7\}}(K)$. It follows that the spanning forests of *K* can be readily constructed from the spanning forests of $K_W$ and $K_R$, which are, of course, structurally identical.

The weight of a graph that is obtained by the joining operation is easily seen to be the product of the weights of the corresponding subgraphs,

$$\begin{aligned} w(F) = w(F_W) \, w(F_R) \,. \end{aligned}$$

Summing over spanning forests, we see that,

$$\begin{aligned} w(\Phi _{\{1,7\} \rightarrow \{6,7\}}(K)) = w(\Phi _{\{6\}}(K_W)) \, w(\Phi _{\{1,7\}}(K_R)) \,, \end{aligned}$$

and by a similar argument,

$$\begin{aligned} w(\Phi _{\{1,6\} \rightarrow \{6,7\}}(K)) = w(\Phi _{\{1,6\}}(K_W)) \, w(\Phi _{\{7\}}(K_R)) \,. \end{aligned}$$

It follows from Eqn. 64 that,

$$\begin{aligned} \eta = \frac{w(\Phi _{\{6\}}(K_W)) \, w(\Phi _{\{1,7\}}(K_R))}{w(\Phi _{\{1,6\}}(K_W)) \, w(\Phi _{\{7\}}(K_R))} \,, \end{aligned}$$

where

$$\begin{aligned} w(\Phi _{\{6\}}(K_W))&= k_{p,W} \left( k_{2,W} k_{-3,W} + k_{1,W} k_{2,W} + k_{-1,W} k_{-3,W} \right) \\&= k_{p,W} \left( k_{2,W} \left( k_{-3,W} + k_{1,W} \right) + k_{-1,W}k_{-3,W} \right) \\ w(\Phi _{\{1,6\}}(K_W))&= k_{-1,W} k_{3,W} + k_{-1,W} k_{-2,W} + k_{2,W} k_{3,W} + k_{-1,W} k_{p,W} + k_{2,W} k_{p,W} \\&= \left( k_{-1,W} + k_{2,W} \right) \left( k_{3,W} + k_{p,W} \right) + k_{-1,W} k_{-2,W} \\ w(\Phi _{\{7\}}(K_R))&= k_{p,R} \left( k_{2,R} k_{-3,R} + k_{1,R} k_{2,R} + k_{-1,R} k_{-3,R} \right) \\&= k_{p,R} \left( k_{2,R} \left( k_{-3,R} + k_{1,R} \right) + k_{-1,R} k_{-3,R} \right) \\ w(\Phi _{\{1,7\}}(K_R))&= k_{-1,R} k_{3,R} + k_{-1,R} k_{-2,R} + k_{2,R} k_{3,R} + k_{-1,R} k_{p,R} + k_{2,R} k_{p,R} \\&= \left( k_{-1,R} + k_{2,R} \right) \left( k_{3,R} + k_{p,R} \right) + k_{-1,R}k_{-2,R} \,. \end{aligned}$$

The reader may check that this expression for $\eta$ is identical to that given by Banerjee et al. (2017, Eqn. S9), which was calculated using Mathematica (Oleg Igoshin, private communication). The methods introduced here allow these calculations to be done by hand and reveal how the structure of the formula arises from the linear framework graph.

## Discussion

The linear framework emerged in Gunawardena (2012) from the fundamental realisation that the steady-state vector of a Laplacian dynamical system, as described by Eqn. 1, may be expressed as manifestly positive rational algebraic functions of the edge labels of the corresponding graph (Eqn. 5), by virtue of the Matrix-Tree theorem (Eqn. 4). This algebraic access to the steady state has been invaluable because it has yielded insights without having to estimate the numerical values of the transition rates, which, in the biological context, are usually unknown. The underlying graph also enables particular system features to be specified, while allowing the structure and the topology of the rest of the graph to remain arbitrary. This has enabled general theorems to be proved that rise above the ever-present molecular complexity that confronts us in biology. The impact of these capabilities has been reviewed in Nam et al. (2022).

The present paper has extended these capabilities to the transient regime in the microscopic setting, where the graph describes the infinitesimal generator of a Markov process and the Laplacian dynamics gives the corresponding master equation. The All-Minors Matrix-Tree theorem (Theorem 1) plays the key role, by showing how the inverse of a principal submatrix of the Laplacian may be calculated in terms of spanning forests (Proposition 2). The splitting probabilities to reach a set of target vertices (Theorem 4), the moments of the conditional FPT to a specific target vertex (Theorem 5) and the moments of the unconditional FPT to any target vertex (Theorem 6) may then be expressed as manifestly positive rational algebraic functions of the transition rates.

These results are timely. Timescale separation, in which a sub-system is assumed to have reached a steady state, has been a convenient approximation for analysing biochemical systems ever since Michaelis and Menten first introduced the method in 1910 (Gunawardena 2014). This has proved to be a good approximation for *in vitro* biochemistry but whether it remains appropriate within living cells is less clear (Wong and Gunawardena 2020). Moreover, as noted in the Introduction, a variety of single-molecule methods have made the transient regime experimentally accessible and FPTs have been widely used to analyse the resulting data (Kou et al. 2005; Shaevitz et al. 2005; Kolomeisky and Fisher 2007; Chemla et al. 2008; Garai et al. 2009; Bel et al. 2010; Moffitt et al. 2010; Moffitt and Bustamante 2014; Banerjee et al. 2017; Cui and Mehta 2018; Mallory et al. 2019; Wang et al. 2021; Lammers et al. 2020; Alamos et al. 2023; Lammers et al. 2023; Dal Co et al. 2017; Ghusinga et al. 2017; Gupta et al. 2018; Ham et al. 2024). Indeed, the most immediate application of the results presented here has been to analysing the interplay between specificity and speed in the CRISPR–Cas9 system, which we report on elsewhere (Nam et al. 2025).

Previous analyses of FPTs in the biological context have not yielded the kinds of closed-form mathematical formulas found in Theorems 4, 5 and 6. Accordingly, it has been difficult to extricate general principles and the underlying mathematical structure of FPTs as rational algebraic functions has been obscured. Our results make clear that, in this respect, the transient regime shares the same mathematical character as the steady state.

Notwithstanding this similarity, many interesting questions remain as to the distinctions between these two regimes. One such question concerns the role of energy expenditure. As noted in the Introduction, the formula for steady-state probabilities in Eqn. 5 reduces to the standard prescription of equilibrium statistical mechanics when the graph satisfies the *cycle condition* and thereby reaches a steady state of thermodynamic equilibrium (Gunawardena 2012; Ahsendorf et al. 2014; Estrada et al. 2016). This reduction has been particularly useful in characterising what we have called *Hopfield barriers*, or the limits to information processing at thermodynamic equilibrium (Estrada et al. 2016; Martinez-Corral et al. 2024). These limits have been studied at steady state but an interesting problem emerges as to whether similar limits may exist in the transient regime. In other words, if a system is relaxing to a steady state of thermodynamic equilibrium, is its capability to process information fundamentally limited, compared to when it is relaxing to a non-equilibrium steady state? Preliminary calculations, on which we hope to report subsequently, suggest that this may be the case. However, such barriers seem to arise in the transient regime for quite different mathematical reasons to their appearance at steady state. For instance, the rational functions for splitting probabilities, in Theorem 4, and for FPT moments, in Theorems 5 and 6, do not appear to simplify in any obvious way when the cycle condition is satisfied, as is the case for steady-state probabilities. It remains a challenging open problem to understand how thermodynamic equilibrium may be influencing the mathematical structure of the rational algebraic formulas introduced here.

The most obvious mathematical distinction between the steady state and the transient regime, in the treatment we have provided here, is that the rational algebraic descriptions of the former arise from spanning trees, while those of the latter arise from spanning forests. This distinction, however, may not be a fundamental one. The pioneering biophysicist, Terrell Hill, suggested a way to express mean unconditional FPTs and splitting probabilities in terms of the steady-state probabilities of a modified Markov process (Hill 1988). (We revisited some of Hill’s calculations in the examples treated above.) Similarly, Mark Kac pointed out that the mean recurrence times for a discrete-time Markov chain can be related to its steady-state probabilities (Kac 1947), and a similar result holds for continuous-time Markov processes (Serfozo 2009). These findings suggest a more systematic relationship between the transient and steady-state regimes and between spanning forests and spanning trees. We explore this further in a sequel to the present paper, in which we prove generalisations of the work of Hill and Kac (Nam and Gunawardena 2025).

The results presented here bring the transient regime of Markov processes within the same rational algebraic perspective that the linear framework previously developed for steady states. We hope this will encourage others to build upon the foundations laid here.

## Data Availability

This paper has no associated data.

## Appendix

**Table 1:** Summary of the notation used in the paper

*General mathematics*	
$\textbf{0}$, $\textbf{1}$	Zero or all-ones matrix or vector
$\textbf{I}$	Identity matrix
$\# A$	Size of the set *A*
$\textbf{M}_{[A,B]}$	Submatrix of $\textbf{M}$ with the rows indexed by *A* and columns indexed by *B*
$\theta _A : \left\{ 1, \dotsc , k \right\} \rightarrow A$	The ordering bijection on $A = \left\{ a_1< \cdots < a_k \right\}$, $\theta _A(i) = a_i$ for $i = 1, \dotsc , k$
$\mathscr {L}\left\{ f \right\}$	Laplace transform of *f*
*Graphs*	
*G*	Graph
$\mathcal {V}(G)$	Vertices of *G*, taken to be $\left\{ 1, \dotsc , N \right\}$ unless otherwise specified
*N*	Number of vertices in *G*, unless otherwise specified
$\mathcal {E}(G)$	Edges of *G*
$i \rightarrow j$, $i \rightarrow _G j$	Edge from vertex *i* to vertex *j* in *G*
$\ell (i \rightarrow j)$, $\ell (i \rightarrow _G j)$	Label on edge $i \rightarrow j$ in *G*, with units of $(\text {time})^{-1}$
${\overline{A}}$	Complement of the vertex subset $A \subseteq \mathcal {V}(G)$
$\mathcal {L}(G)$	Laplacian matrix of *G* (Eqn. 2)
$\textbf{L}(G)$	$-\mathcal {L}(G)^\textrm{T}$ (Eqn. 41)
$\lambda _i$	Sum of outgoing edge labels from *i*
$\textrm{V}_{\text {out}}(i)$	Vertices to which there is an edge from *i*, $\left\{ j \in \mathcal {V}(G) : i \rightarrow j \right\}$
$\textrm{V}_{\text {in}}(i)$	Vertices from which there is an edge to *i*, $\left\{ j \in \mathcal {V}(G) : j \rightarrow i \right\}$
$i \leadsto j$, $i \leadsto _G j$	Existence of a directed path of edges from *i* to *j* in *G*
$\mathcal {V}_{\text {out}}(i)$	Vertices to which there is a directed path of edges from *i*, $\left\{ j \in \mathcal {V}(G) : i \leadsto j \right\}$
$\mathcal {V}_{\text {in}} (i)$	Vertices from which there is a directed path of edges to *i*, $\left\{ j \in \mathcal {V}(G) : j \leadsto i \right\}$
$\mathcal {V}_{\text {in}}^{-}(i)$	$\mathcal {V}_{\text {in}} (i) \setminus \{i\}$
$w(\cdot )$	The weight, or product of edge labels, of a subgraph, or the sum of those for a set of subgraphs
$\mathcal {C}(G)$	Strongly connected components (SCCs) of *G*
$\mathcal {T}(G)$	Terminal SCCs of *G*
*T*	Number of terminal SCCs in *G*
$G^\sigma$	Graph obtained from *G* by re-indexing the vertices according to a permutation, $\sigma : \mathcal {V}(G) \rightarrow \mathcal {V}(G)$
$\tau _{\sigma ,A}$	Given $A \subseteq \mathcal {V}(G)$ and a permutation $\sigma : \mathcal {V}(G) \rightarrow \mathcal {V}(G)$, the permutation $\theta _{\sigma (A)}^{-1}(\sigma (\theta _A))$ on $\left\{ 1, \dotsc , \#A \right\}$ (Eqn. 14)
*Z*	If *G* is a graph with terminal vertices (singleton terminal SCCs), the union $\bigcup {\mathcal {T}(G)}$; taken to be $\left\{ N-T+1, \dotsc , N \right\}$ without loss of generality
$\mathcal {Z}(i)$	Subset of terminal vertices *z* such that $i \leadsto z$ (Eqn. 31)
$N_z$	Number of non-terminal vertices that lead to the terminal vertex *z*
*Spanning trees and forests*	
$\mathcal {R}(F)$	Set of roots of a spanning forest *F*
$\Phi _A(G)$	Set of spanning forests of *G* rooted at $A \subseteq \mathcal {V}(G)$
$\Phi _{B \rightarrow A}(G)$	Assuming $\# A = \# B$, the set of spanning forests of *G* rooted at $A \subseteq \mathcal {V}(G)$ in which each $b \in B \subseteq \mathcal {V}(G)$ has a path to a distinct root in *A*
$\chi _F : B \rightarrow A$	Given a spanning forest $F \in \Phi _{B \rightarrow A}(G)$, the bijection $B \rightarrow A$ that maps each $b \in B$ to the root in *A* to which it has a path (Theorem 1)
$\eta : A \rightarrow B$	Given vertex subsets $A, B \subseteq \mathcal {V}(G)$ of the same size, the bijection $A \rightarrow B$ that maps the *i*-th vertex in *A* to the *i*-th vertex in *B* (Theorem 1)
*Markov processes*	
$X(\cdot )$	Markov process associated with *G*
$p_{i,z}(t)$	Probability that $X(\cdot )$ reaches the terminal vertex *z* by time *t* (Eqn. 25)
$\pi _{i,z}$	Splitting probability from *i* to *z* in *G* (Eqn. 26)
$p_{c,i,z}(t)$	Probability that $X(\cdot )$ reaches the terminal vertex *z* by time *t*, conditioned on *z* being reached (Eqn. 27)
$p_{i,Z}(t)$	Probability that $X(\cdot )$ reaches some terminal vertex by time *t* (Eqn. 29)
$u_{i,z}(t)$	Density corresponding to $p_{i,z}(t)$ (Eqn. 35)
$\mu _{i,z}^{(r)}$, $\mu _{c,i,z}^{(r)}$, $\mu _{i,Z}^{(r)}$	*r*-th moments of $p_{i,z}(t)$ (Eqn. 43), $p_{c,i,z}(t)$ (Eqn. 50), and $p_{i,Z}(t)$ (Eqn. 53)
